# An estimator of first coalescent time reveals selection on young variants and large heterogeneity in rare allele ages among human populations

**DOI:** 10.1371/journal.pgen.1008340

**Published:** 2019-08-19

**Authors:** Alexander Platt, Alyssa Pivirotto, Jared Knoblauch, Jody Hey

**Affiliations:** Center for Computational Genetics and Genomics, Dept. Biology, Temple University, Philadelphia, Pennsylvania, United States of America; University of California, Los Angeles, UNITED STATES

## Abstract

Allele age has long been a focus of population genetic research, primarily because it can be an important clue to the fitness effects of an allele. By virtue of their effects on fitness, alleles under directional selection are expected to be younger than neutral alleles of the same frequency. We developed a new coalescent-based estimator of a close proxy for allele age, the time when a copy of an allele first shares common ancestry with other chromosomes in a sample not carrying that allele. The estimator performs well, including for the very rarest of alleles that occur just once in a sample, with a bias that is typically negative. The estimator is mostly insensitive to population demography and to factors that can arise in population genomic pipelines, including the statistical phasing of chromosomes. Applications to 1000 Genomes Data and UK10K genome data confirm predictions that singleton alleles that alter proteins are significantly younger than those that do not, with a greater difference in the larger UK10K dataset, as expected. The 1000 Genomes populations varied markedly in their distributions for singleton allele ages, suggesting that these distributions can be used to inform models of demographic history, including recent events that are only revealed by their impacts on the ages of very rare alleles.

## Author summary

We developed a way to estimate the time when a copy of a gene most recently shared ancestry with other copies of that gene. This is also an estimate of the upper bound of when a mutation has arisen, and it can be used to study the ages of alleles that are found in a population. The method can be applied to the very rarest alleles found only once in a sample, even in studies of many thousands of genomes. We tested the method extensively, found it performs well, and can be used under a wide variety of conditions. We applied it to 1000 Genomes project data (26 populations) and the UK10K data (over 7000 genomes) and found clear evidence that alleles that change proteins are younger than alleles that do not, as expected. We also observed wide variation in the ages of alleles at low frequency among the 1000 Genome project populations, indicating that our method could be used to study the demographic history of human populations. Going forward, the estimator should be useful for many kinds of questions in population genomics, particularly as sample sizes continue to grow.

## Introduction

The age of an allele of a given frequency can be reveal the forces acting upon it, with rare alleles being particularly sensitive to recent evolutionary processes [1]. A functional allele that is younger than expected given its frequency is likely to have been under directional selection. This is not surprising for favored alleles, but it is also true for harmful alleles [2], including those with negative impacts on health that are under negative selection [3]. If researchers are able to estimate allele age, they could combine this with other information (e.g. allele frequency, geographical distribution, functional annotation) to improve predictions of an allele’s effect on human health. Alternatively, an allele that is older than expected given its frequency is also a candidate for having an interesting history, as functional alleles older than expected can be the result of balancing or negative frequency-dependent selection [4].

As population genomic samples grow in size, the density of variable sites rises approximately in proportion to the log of the sample size [5], and very large data sets will have large numbers of SNPs and other variants in every gene. If information could be gleaned on the ages of a large number of variants for a functional region of the genome, this could be used to develop a detailed portrait of the history of natural selection specifically on that region.

Allele ages are also shaped by processes that act in aggregate across the genome. The overall distribution of ages will be strongly shaped by the demographic history of the population, and for the rarest alleles, that distribution will be acutely sensitive to recent admixture [6]. The age spectrum will also fluctuate spatially along the genome, both stochastically and as a function of the intensity of background selection [7].

In developing a way to study allele age, we considered several constraints. An estimator should not be a function of allele frequency, as we wish to glean information about allele history that is distinct from its frequency. We also prefer an approach that is not a function of the demographic history of the population, as some estimators are [8, 9]. An estimator that can get close to the true value of the unknown, regardless of demographic history, enables analyses in cases when the history is not known and it enables comparisons between populations that are not confounded by errors in our knowledge of the demographic history of the populations. We also wish to be able to study the age of the very rarest alleles, including those that appear only once in a sample (singletons). This last criterion leads to an approach that is different from existing methods that are based on the variation observed among copies of an allele, or in flanking regions [8–13]. An estimator should also be applicable for very large sample sizes for which it becomes increasingly possible to find low frequency alleles that arose by multiple mutations [14]. For these cases, as with singletons, we need a method that is applicable for each individual gene copy. Finally, an estimator should not be highly sensitive to the details of the bioinformatics pipeline used to process the data, such as whether the data were statistically phased.

We developed a new estimator that focuses, not directly on allele age, but rather on the time when a base position in a particular chromosome first coalesces in the genealogy. The mutation causing a derived allele at that base will have occurred since this first coalescent time, and so first coalescent time can be used as a proxy for allele age. For example, a neutral singleton allele will have a uniform probability of having arisen anywhere along an external branch, and therefore an expected age of half the first coalescent time. We assessed performance using simulated data, and show that it performs well and substantially overcomes the challenges describe above. We also applied it to SNP alleles from the 1000 Genomes Project [15] and the UK10K genome panel [16]. For these data sets, we assessed basic predictions regarding population-specific variation in the ages of rare alleles, the ages of private alleles and alleles shared by populations, and we compared ages of alleles that are expected *a priori* to have functional impacts with those that are not. Our estimator shares structural similarities with existing methods developed to estimate demographic histories from shared-haplotype tract-length distributions [17–20], but is uniquely able to discern the specific histories of individual rare alleles.

## Results

### An estimator of first coalescent time

**Fig 1:**
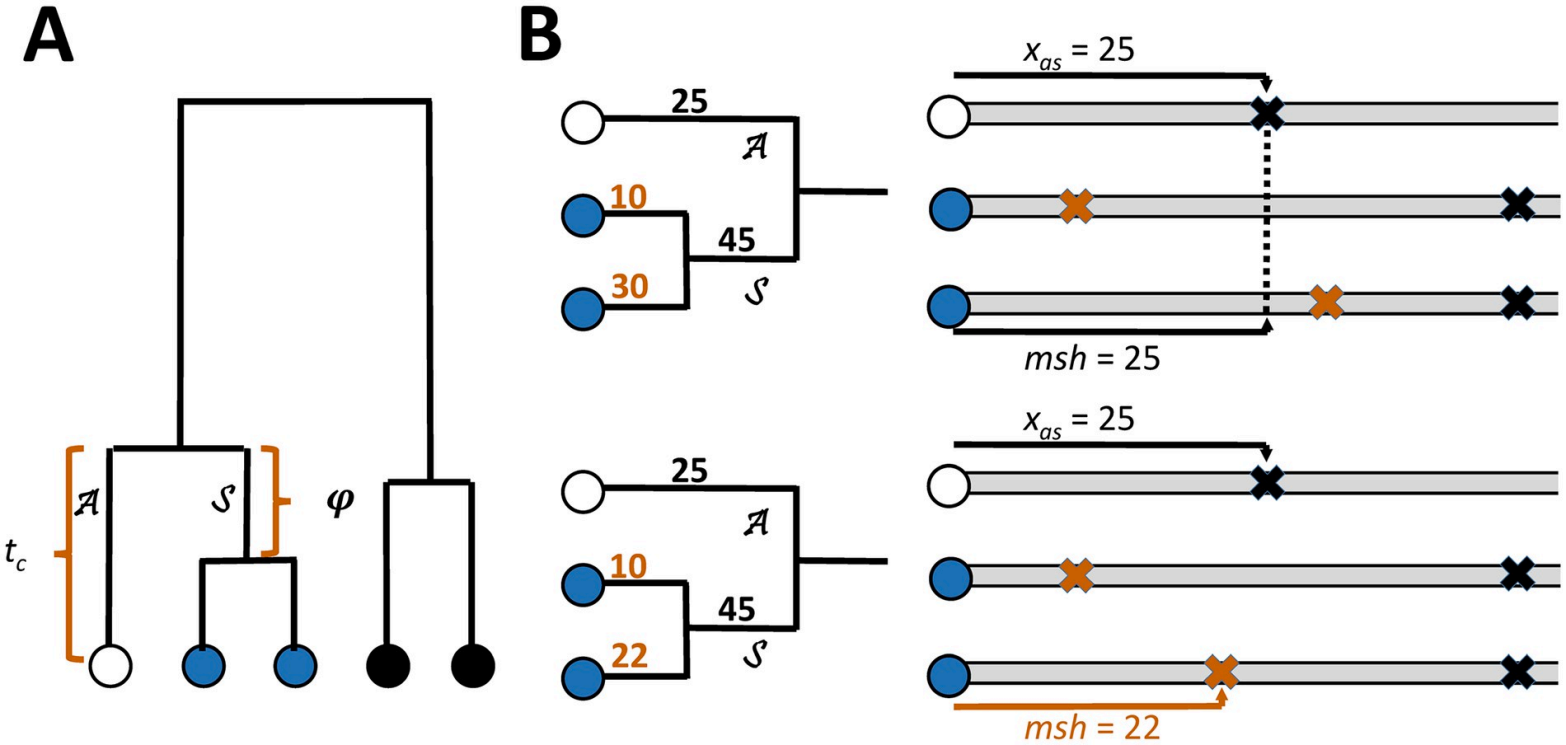
**A**. Gene tree showing a chromosome at the focal base (white) as the terminus of branch A, which has length *t*_*c*_, and a sister clade (blue) which share a branch S of length *φ*. **B**. Two situations regarding the maximum shared haplotype (*msh*) and the closest mutation on either branch A or $S\left(x_{AS}\right)$. Each is depicted as a genealogy (left side) and an adjacent alignment of the focal chromosome with its sisters (only the alignment to the right side of the focal base is shown). Each branch is labeled with the distance from the focal base position to the most proximal mutation to arise on that branch. Numbers in black are distances to events that may contribute to both *msh* and *x*_*AS*_. Numbers in brown are distances that can only contribute to the observed *msh* but not *x*_*AS*_ as they involve events that did not happen on branch A or S. Each mutation is shown as an X mark in the corresponding chromosome alignment. Top panel: Not all sister chromosomes have mutations closer than the mutations on A or S, thus *msh* = *x*_*AS*_. Bottom panel: *msh* is less than *x*_*AS*_ as both sister chromosomes have independently acquired mutations closer to the focal locus than mutations on branches A or S.

Consider one chromosome (the focal chromosome) from a sample of chromosomes drawn at random from a population, and an individual base position on that chromosome (the focal base). As shown in Fig 1, the focal base can be thought of as the terminal point on a branch A of the genealogy of the sample of chromosomes at that base position. If the focal base is a singleton variant (i.e. a derived allele that occurs only once in the sample), then the mutation causing that allele must have occurred on this branch. Also shown in Fig 1 is that branch A connects the focal chromosome to the most recent common ancestor of that chromosome and a sister branch S, which is ancestral to one or more sister chromosomes of the focal chromosome. Branches A and S share their most recent common ancestor at a time *t*_*c*_ generations prior to the sample generation, and branch A is therefore *t*_*c*_ generations long. We denote the length of branch S as *φ* generations. If the sample contains a single unique sister chromosome (that is, the focal chromosome is also the closest relative of the sister at the focal base), then *φ* is equal to *t*_*c*_. When multiple chromosomes are all equally and most closely related to the focal chromosome at the focal base, they form a clade of sisters, and *φ* < *t*_*c*_.

Consider first the case when there is no recombination and the focal chromosome has just a single sister. In this situation, any differences between the focal and sister chromosomes will have been caused by mutations on branches A and S. We model mutation as a Poisson process, where each base has a constant probability, *μ*, of mutating in each generation. Treating a chromosome as a continuous line, and considering events just to one side of the focal base (in either the 5’ or 3’ direction), the probability density of distance *x* from a focal base to the first base that is not identical between the two chromosomes can be approximated with an exponential distribution having a rate of *μ*2*t*_*c*_:

$$p(x)={\mu 2t_{c}e}^{-x\mu 2t_{c}}.$$ (1)

If we knew which chromosome was the sister chromosome, we could compare them and identify the distance from the focal base to the nearest difference between the chromosomes (i.e. *x*), and use this to estimate *t*_*c*_. This basic idea captures the underlying rationale of our approach. The final formulation takes into account the remaining issues: that we do not know which chromosome is the sister to the focal chromosome; that the focal chromosome may have multiple sisters; and that Eq (1) assumes no recombination (see Materials and methods). Ultimately, an expression that resembles Eq (1), but that differs in replacing *x* with the longest observed tract of identity between the focal chromosome and each of the other chromosomes in the sample, proves applicable. We call this quantity the maximum shared haplotype (*msh*), and show that it arises on either branch A or S (Fig 1) with high probability.

### Estimator performance

We use ${\hat{t}}_{c}$ to denote the estimator, and because *t*_*c*_ values range over several orders of magnitude and Poisson processes have variances proportional to their means, we focused primarily on the logarithm, ${log}_{10}({\hat{t}}_{c})$. Performance was assessed in terms of the root mean squared error (RMSE), bias (mean of estimated minus true values), and correlation (Pearson’s *r* for the true and estimated values of *log*_10_(*t*_*c*_)) for alleles at all frequencies in a series of large simulated data sets. We varied sample size and recombination rate, and considered three demographic histories that varied in terms of population sizes, exponential growth, historical bottlenecks, and intercontinental migration. We also considered samples of chromosomes with known and with estimated phase. All of these results are summarized in S1 and S2 Tables. For low and intermediate recombination, over a wide range of circumstances, the estimator exhibits an RMSE of about 0.4 log-transformed generations, corresponding to estimates that are typically within a factor of 2.5 of the true value. Across the models, correlations of true and estimated values ranged from 0.4 to 0.95 with a mode of 0.9. For recombination rates equal to or less than the mutation rate, bias varies from -0.4 to 0.1 with a mode of -0.2, which corresponds to an average underestimate by a factor of 0.63. Performance was consistent across the spectrum of allele frequencies, and with respect to particular independent variables, performance was better: when recombination was low; when population size was constant; when phase was known; and when sample sizes were larger. A key factor that determines how informative estimates are is having chromosomes that are much more closely related to their closest relatives than to unrelated chromosomes. Thus, strong recent growth can produce more star-like genealogies that reduces these differences and reduce the quality of the estimates, while larger sample sizes improve them.

When recombination rate is appreciably higher than the mutation rate, such that typical *msh* values are small enough to be within the range of distances between average pairwise SNPs, estimates worsens and the bias shifts from negative to positive (S2 Table). The amount of recombination that is too high relative to the mutation rate will depend on the length of the region of high recombination (e.g. if it is associated with hotspots of short length), and on sample size and the demographic history of the sample. We observed that larger sample sizes exert a greater improvement on the quality of estimates in the case of high recombination than with low to intermediate recombination (S1 and S2 Tables). The change in sign of the bias, with high recombination, and that the observed absolute value of the bias is lower with intermediate levels of recombination, suggests that there are multiple contributions to the bias.

**Fig 2:**
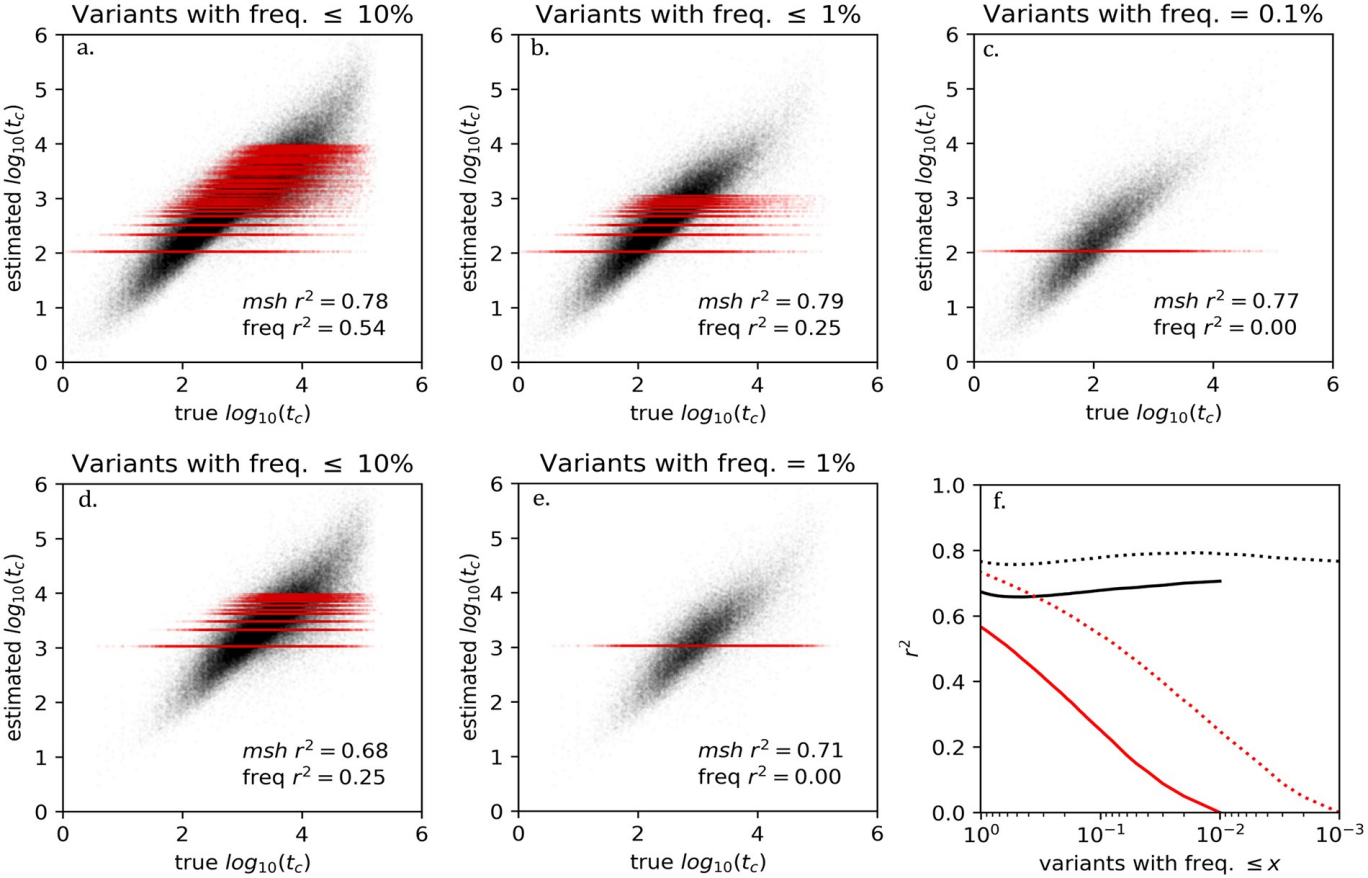
Panels **a** thru **e**. Shown in black are estimated *log*_10_(*t*_*c*_) values plotted against true values from simulations of 1000 (top row) or 100 (bottom row) chromosomes. For comparison are shown values in red for a hypothetical estimator of *log*_10_(*t*_*c*_) that is based only on allele frequency (all alleles at a given frequency will share the same estimated value of *log*_10_(*t*_*c*_). For a given allele frequency *k* the hypothetical frequency-based estimate is a value *τ*_*k*_ that minimizes the sum of squared residuals with respect to the true *Log*_10_(*t*_*c*_) values, i.e. ∑_*i*_(*τ*_*k*_−*Log*_10_(*t*_*c*,*i*_))^2^ where variant *i* with frequency *k* has true first coalescent time *t*_*c*,*i*_. **f**. The square of Pearson’s correlation coefficient is plotted against allele frequency for both estimators, for sample of 1000 (dotted lines) and 100 chromosomes (solid lines).

Fig 2 shows plots of estimated versus true values for different ranges of allele frequencies and for two different sample sizes (box plots are shown in S1 Fig). Also shown in Fig 2 is an idealized estimator of *log*_10_(*t*_*c*_) that is based on allele frequency and for which all alleles at a given frequency generate the same estimate–shown as a band of red points. We show both the *msh*-based and the idealized frequency-based estimators to highlight the contrast; the former does not make use of information about a variant’s frequency while the latter does not make use of any information about a variant’s *msh* value. Unlike the *msh*-based estimator, where each variant may have a unique *msh* from which to generate a unique *t*_*c*_ estimate, no frequency-based estimator can distinguish among the potentially large number of variants occurring at identical frequencies within a sample. When considered over the full range of allele frequencies, the idealized estimator can explain nearly as much of the variation in *log*_10_(*t*_*c*_) as the *msh*-based estimator (Fig 2f). However, for rare alleles, the *msh*-based stimator retains strong performance, whereas the frequency-based estimator explains little to none of the variation in *log*_10_(*t*_*c*_).

### Phasing

**Fig 3:**
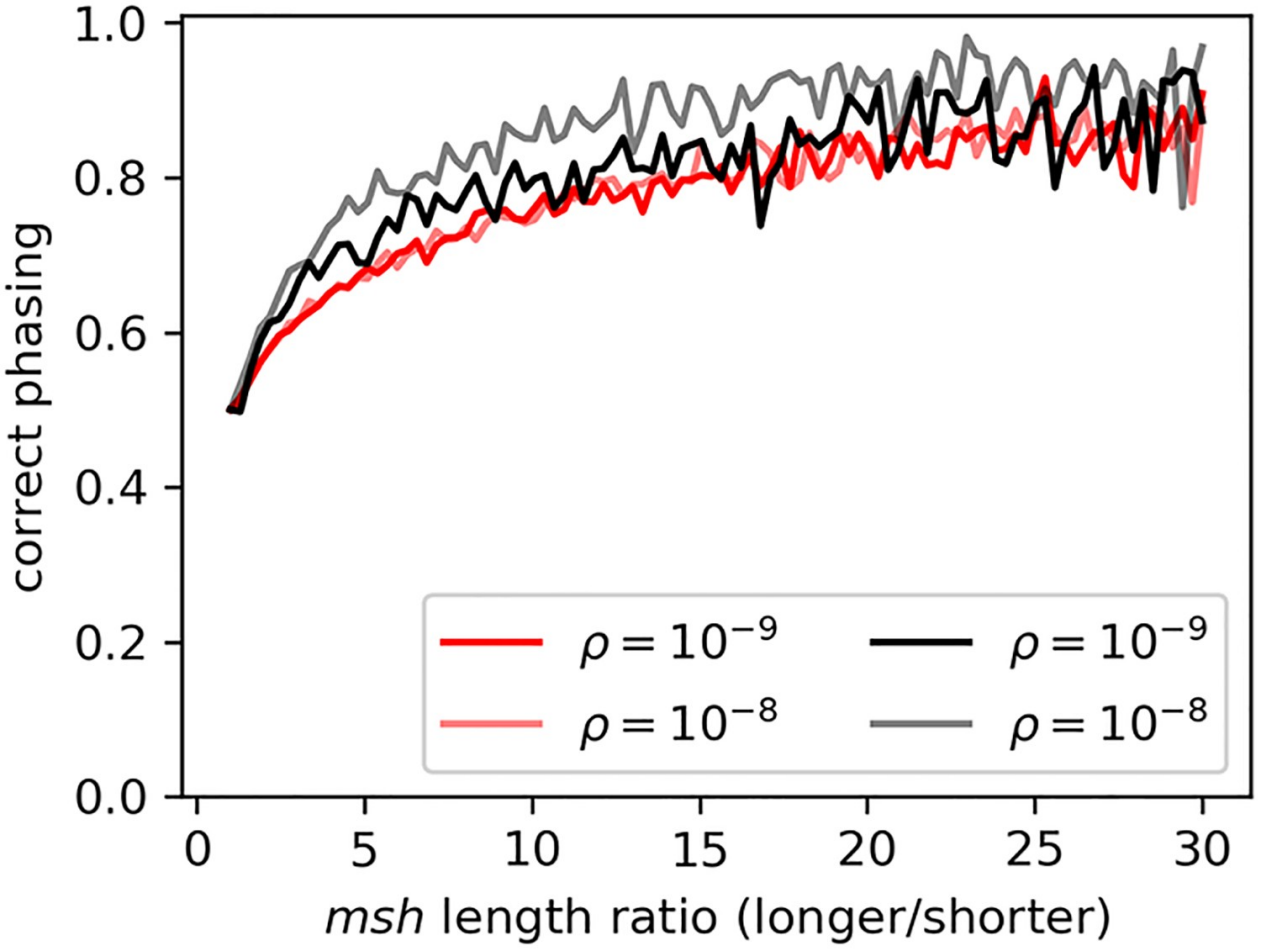
Frequency of a singleton variant residing within the shorter of two *msh* tracts. Accuracy of phasing singleton variants identified in simulations of *n* = 1000 (red) and of *n* = 100 (black) in 100 equal-width bins of the ratio of the longer to shorter *msh* tracts of the genotype carrying the rare variant.

An important application is assessing upon which of the two chromosomes of an individual a singleton allele correctly resides. As shown in Fig 3, the phasing accuracy for singleton variants in simulated data rises as the ratio of the genetic lengths of the alternative *msh* tracts diverge. For variants with *msh* tracts of similar length (and thus high probability of misassigned phase), similar ${\hat{t}}_{c}$ values will be found regardless of how phase is assigned.

For non-singleton variants, the estimator is expected to be relatively immune to switch errors introduced by haplotype phasing software. This is because switch errors typically involve low frequency variants among similar haplotypes [21, 22], and these do not typically affect the distribution of *msh* values which are often terminated by relatively common variants or recombination events between unrelated haplotypes. S2 Fig shows that results for statistically phased chromosomes are quite similar to those for the correct chromosomes (*r* = 0.941) based on analyses of singleton alleles in male X-chromosome UK10K data.

From S1 and S2 Tables we see that mean error is slightly increased when the data (including singletons) are phased statistically. The bias also becomes more negative by a small amount with phasing. Singletons are phased by assigning them to the chromosome that reveals the shorter *msh* and thus the longer ${\hat{t}}_{c}$. The proximal effect of this will be to introduce a positive bias that applies in those instances when this phase assignment is not correct. However, the observation that bias becomes slightly more negative with phasing, including for singletons, suggests a greater effect in the other direction. It is possible that the phasing of singletons prior to determining *msh* values causes them to cluster on fewer chromosomes, thereby lengthening the *msh* values that are observed.

### 1000 Genomes Project analyses

**Fig 4:**
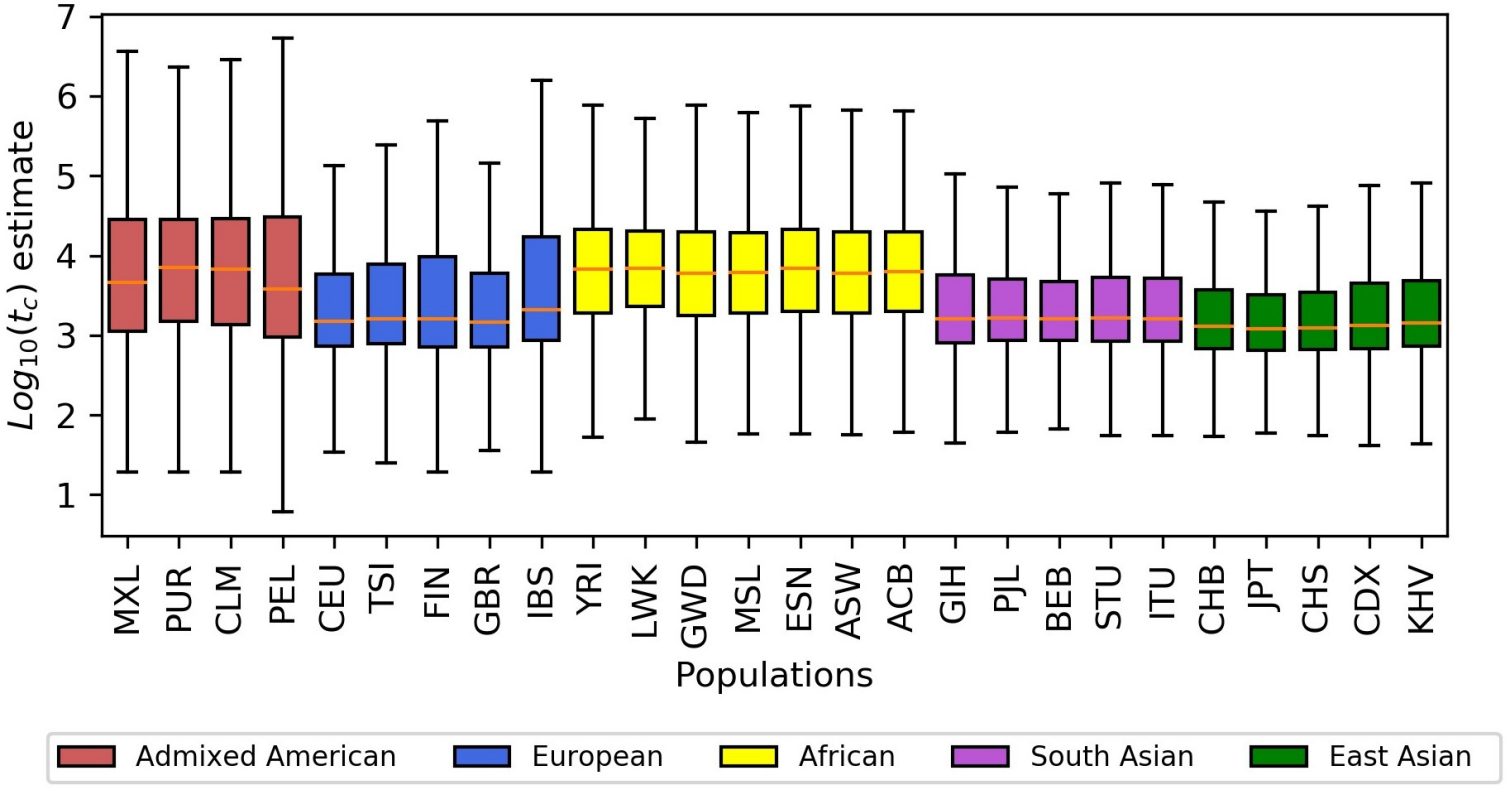
Distributions of *log*_10_(${\hat{t}}_{c}$) values for singleton variants from each 1KGP population.

Based on analysis of variation (ANOVA, Table 1, S6 Table), we learned that different populations show different distributions of ${log}_{10}({\hat{t}}_{c})$ values for singleton variants (*d*. *f*. = 25; *F* = 19248; *p* < 1 × 10^−128^). As shown in Fig 4 and S3 Table, populations in Africa have higher geometric means (6353 to 7178 generations) than populations from regions that have not had substantial admixture from African populations (East Asia, South Asia, and Europe), which have lower geometric means (2018 to 3882 generations). Admixed American populations spanned a wide range (5610 to 7194), with values near those of the African populations. The finding of younger ages for singletons from non-African old-world populations is expected under a general Out-of-Africa model in which those populations have passed through a bottleneck and have had an overall lower effective population size and thus shorter coalescent times.

**Table 1:** Type-II ANOVA results for *log*_10_(${\hat{t}}_{c}$) values for 1KGP singletons, where *PC* is a categorical variable indicating protein-changing status, *Private* is a categorical variable indicating the restriction of a variant to a single population, and *Population* is a categorical variable indicating the population in which a variant is observed.

	Sum of squares	Degrees of freedom	*F*	Pr (> *F*)
*Population*	2.61E+05	25	19248.54	0
*PC*	1.30E+01	1	23.93626	9.96 × 10^−7^
*Private*	4.86E+05	1	896297.7	0
*PC* × *Population*	7.93E+00	25	0.584825	9.5 × 10^−1^
*PC* × *Private*	1.55E+00	1	2.86222	9.07 × 10^−2^
*Private* × *Population*	4.66E+04	25	3437.326	0
*Residual*	4.67E+06	8611081	--	--

**Fig 5:**
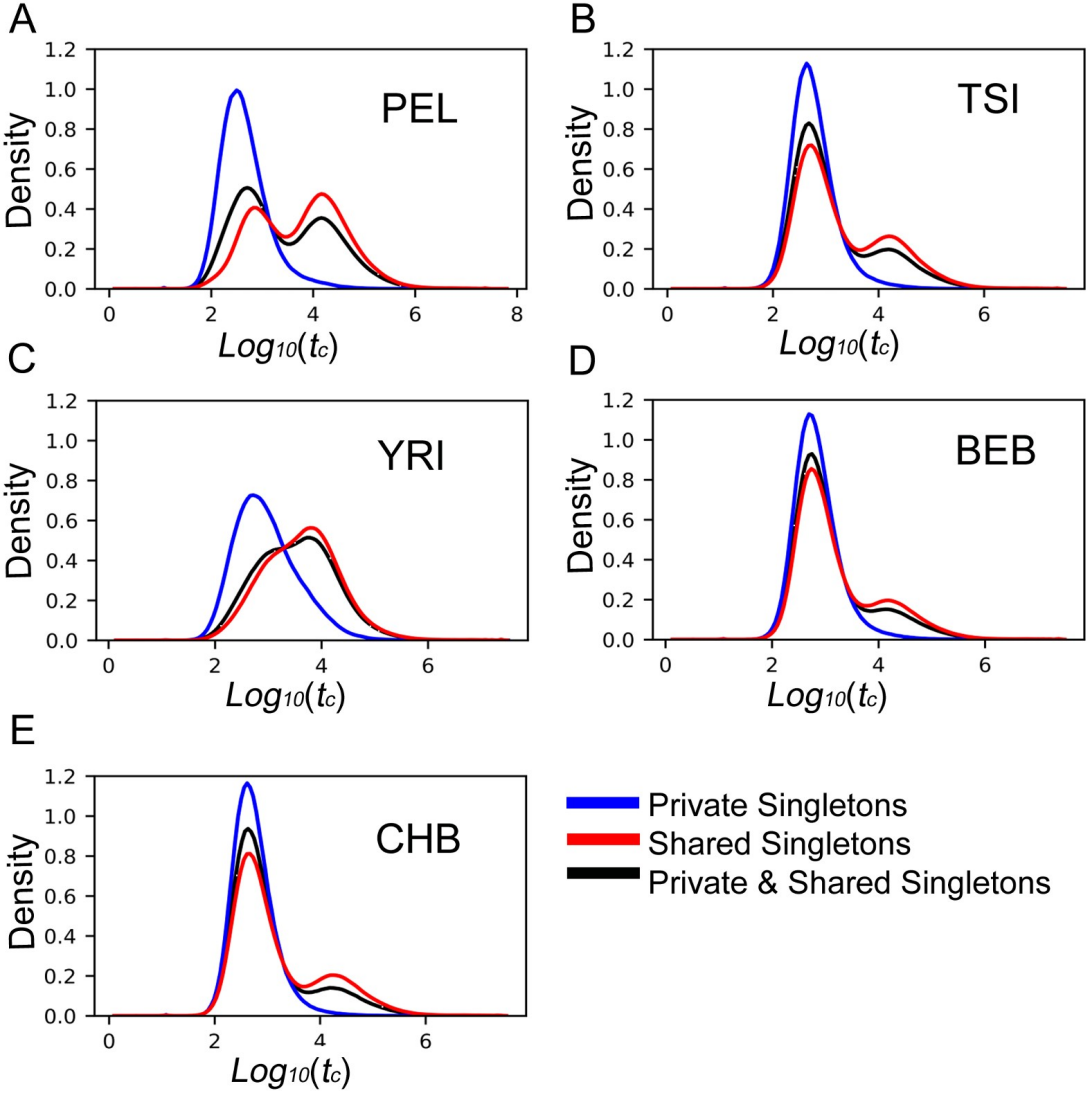
Distributions of ${log}_{10}({\hat{t}}_{c})$ values for 1% frequency alleles (singletons) from representatives of five super-populations. Shown in each panel are distributions for alleles found only in that population at 1% (Private), alleles that are at 1% in that population, but also shared with one or more other populations (Shared), and the sum of Private and Shared. A. the Peruvian population(PEL) from the admixed American super-population. B. the Turin population (TSI) from Europe. C. the Yoruban population (YRI) from Africa. D. the Bengali population (BEB) from South Asia. E. the Bejing Han Chinese population (CHB) from East Asia.

The 1KGP data allow us to compare the age of rare variants that are found only in a single population to the age of variants that have the same low frequency within that population, but that also are found in one or more other populations. For all of the 1KGP populations, private singletons had distributions that were shifted to the left (younger) relative to singletons that were shared. Fig 5 shows these distributions for a single population that is representative of those observed from each the five super-populations, each of which showed a characteristic distribution (S9–S13 Figs). In every population, private singleton alleles are younger than singleton alleles that are also shared with other populations (*d*. *f*. = 1; *F* = 896298; *p* = 0, Table 1), with private singleton variants being 70% younger than comparable shared variants. However, the size of the reduction varies considerably by population (*d*. *f*. = 25; *F* = 3437; *p* = 0, Table 1), with the largest reduction observed in admixed American populations (82% to 92%).

### The age of protein-changing alleles

With respect to the functional impact of rare alleles, we expect that the rarest variants in a sample will be enriched (relative to more common variants) for alleles that have an impact on fitness, and therefore that rare alleles will be targeted more by natural selection. This is because such alleles pass through frequency space more quickly than neutral alleles and, if not lost from the population, will reach a given frequency more quickly than a neutral allele [2]. Consistent with this prediction we observed in the 1KGP data that, conditional on population and geographic spread, singleton variants that alter proteins are 3.6% younger than those that do not (*d*. *f*. = 1; *F* = 24; *p* = 1 × 10^−6^, Table 1). There is no significant difference to the effect of protein-changing status in individual populations (*d*. *f*. = 25; *F* = 0.58; *p* = 0.95, Table 1), and there is no significant interaction between protein-changing status and a variant being private to its population (*d*. *f*. = 1; *F* = 2.86; *p* = < 9 × 10^−2^, Table 1).

The theory that says that alleles that affect fitness will be younger applies to both harmful and beneficial alleles [2]. However, if it is the case that harmful rare alleles are much more common than beneficial rare alleles, then the age difference between functional alleles and neutral alleles should be greater the rarer are the alleles under comparison. This is because most harmful alleles are rapidly removed from a population and are more likely to be observed at the lowest frequencies in larger samples. We therefore predict that we should observe a greater difference in ages between alleles that change proteins and those that do not in the larger UK10K dataset than in the 1KGP data. In fact, the singletons in the 7,242 samples of UK10k data showed a considerably larger effect of protein-changing status than those identified as singletons in the 100-sample populations of the 1KGP data. The geometric mean ${\hat{t}}_{c}$ value for the 10^5^ protein-changing singleton variants was 12% lower than it was for the 1.5 × 10^7^ non-protein-changing variants (305 generations vs 347 generations; Wilcoxon-Mann-Whitney *p* = 0).

## Discussion

Every mutation can be envisioned as occurring on a branch of the genealogy or gene tree for a sample of genomes at the locus where the mutation occurred. Previous estimators of allele age have focused on the time point at which different copies of an allele coalesce with each other, i.e. the time of the top of the gene tree edge upon which the mutation occurred [8–12]. Here we have taken a different approach and focused on the time at the bottom of that same edge, the time of first coalescence for an edge carrying a mutation. These first coalescent times have direct connections to *msh* values, which are easily measured from aligned genomes. The resulting estimator is not without noise, but estimates covary roughly linearly with true values, with moderate error and bias over a wide range of circumstances.

Our estimator also meets a variety of desired criteria established at the outset. It is not a function of allele frequency, and thus can be used to study how an allele came to reach its observed frequency. It is only very weakly a function of demographic history, and thus it can be used to compare the ages of alleles of different populations that have unknown or widely varying histories. It can be applied to alleles that occur only once in a sample, and to genomic data with very large sample sizes. It can be applied individually to each copy of an allele, and thus can be used in cases when an allele has arisen by multiple mutations. And it suffers little degradation in performance when run on statistically phased chromosomes.

The estimator is not expected to be highly sensitive to some additional issues that can arise with population genomic data, including sequencing errors. The UK10K data for example are low coverage (7x), however, because new singleton variants are only called when the data are strongly supportive (high false negative rate, low false positive rate) only a small minority of the singletons in the data set are expected to be errors. In fact, the proportion of called variants in the case of the UK10K sample that are true variants was estimated to be quite high (~94% for singleton variants), based on measurements across monozygotic twins. This is shown in the first figure of the extended data for that paper (panel k, row AC = 1, MZ twins section, column "non-ref genotypes %", divided by two and subtracted from 100) [16]. However the variants that are missing from low coverage data can affect the distribution of *msh* values in ways not accounted for by the method. For example, many of the mutations that terminate tracts of identify and determine *msh* are themselves singletons, and so a coverage bias against singletons will tend to lengthen *msh* values and reduce ${\hat{t}}_{c}$ values.

Although the method can be used to study selection, implicit in the method is an assumption of neutrality, such that mutations on branches A and S (Fig 1) do not affect the distribution of *t*_*c*_ values. But of course a significant fraction of the mutations in evolutionarily constrained regions are not neutral, and the *t*_*c*_ values in these regions are reduced, as we see in our ANOVA results. The question then is, how does this kind of variation in the non-deleterious mutation rate affect *msh* and ${\hat{t}}_{c}$ values? A closely related question applies to factors such as variation in background selection and flucations in rates of gene flow or admixture, that cause polymorphism levels to wax and wane across chromosomes, and that alter the distribution of *msh* values that terminate due to recombination events. The basic expectation is that these kinds of variations will have a greater effect on long *t*_*c*_ values, when flucations in the detminants of what should be short *msh* values can have a greater affect. However short *t*_*c*_ values are associated with very long *msh* values that span large portions of chromosomes and can be expected to be more immune to local flucations in the factors that terminate *msh* tracts.

Another potential difficulty is that as sample sizes grow very large, some rare alleles will have been caused by multiple mutations [14]. While this is not a concern for singletons, it is possible that, for example, a doubleton will actually represent two mutations. In these cases, the likelihood estimator will simply generate a composite of two different singleton coalescent times. Unlike methods that estimate the time of most recent common ancestry among individuals carrying a rare variant, ${\hat{t}}_{c}$ is the estimated time since each copy of the rare variant last shared a common ancestor with an individual *not* carrying the variant. If two copies of an allele are actually the result of independent mutations, the composite ${\hat{t}}_{c}$ estimation will produce an intermediate value and not an aberrantly large one, as would occur with a method based on intra-allelic differences. S3 Fig illustrates the impact of analyzing two independently derived minor variants as copies of a single mutation.

Some additional and important benefits of the estimator described here emerged during the course of this study. Estimated *t*_*c*_ values, or *msh* values, can be used to phase singleton alleles (i.e. estimate the correct chromosome for placement in diploid heterozygous individuals). Another benefit is that the method can be applied relatively quickly to very large data sets with the benefit of the PBWT algorithm [23]. Computing time increase linearly with both sample size and numbers of variants, and all the singleton variants for the full UK10K panel for chromosome 22 can be analyzed in under 30 minutes, for example (S4 Table). A third likely benefit, that has not been explored here, is that it should be possible to work with genomic data in which only dispersed portions of the genome have been sequenced. In particular, *msh* tracts for rare alleles from large samples are typically much longer than the distances between genes, and consequently it should be possible to apply the method to data sets of aligned exome sequences.

Our analysis of variants of 1% frequency among 26 populations of the 1KGP data, revealed considerable systematic variation in allele ages depending on the variant’s function, location, and geographic distribution. Different human populations have variants at 1% frequency with very different distributions of ages. Population-specific demographic histories, including population bottlenecks, expansion, and admixture events, have likely all contributed to these broad differences, and therefore changing, in effect, what it means to be a 1% frequency variant on a population-by-population basis.

We also observed that the ages of variants found at 1% frequency in one population depend greatly on whether or not they are also found in other populations. To be found in multiple populations, a variant that arose by a single mutation must have either: 1) originated prior to the divergence of those populations and risen to sufficient frequency that it has not been lost in either; or 2) persisted in its population of origin long enough and risen to sufficient frequency that migrants have had an opportunity to bring it to at least 1% frequency in another population. Both of these phenomena are reflected in substantial differences of ages of variants found in a population at 1% frequency depending on their total geographic range.

The survey of singleton ${\hat{t}}_{c}$ distributions for 1KGP revealed bimodal distributions in each of the non-African populations (S9–S13 Figs). All of them exhibit a large peak at less than a thousand generations, and another smaller one at more than ten thousand generations, a pattern that is readily interpreted in terms of an older bottleneck associated with the out-of-Africa history of these populations. S14 Fig shows these same patterns arise for simulated data generated with parameter estimates for an Out-of-Africa model [24]. The younger larger peak is characteristic of variants that arose after the population’s ancestors had emigrated from Africa. These are relatively new variants that have not yet had an opportunity to rise to high frequency or spread to other populations. The older peak consists of variants that arose within an ancestral African population and predate the modern human expansion into Eurasia. Although they are identified as singletons in individual populations, these variants are often shared across multiple populations, and they typically occur at frequencies greater than 1% in some populations. In the populations where they are ascertained as singletons, founder events, genetic drift, and natural selection have made these older alleles rare, or even eliminated them entirely before re-introduction by migration from other populations.

Independently of geography and demographics, protein-changing variants in the 1KGP populations that are found at 1% frequency are younger on average than comparable variants that do not change proteins, consistent with previous reports [25]. These are expected to be mostly deleterious variants that have not yet been removed from the population, but they may also include some beneficial variants that have not yet been pushed to higher frequencies. At the 1% frequency level, the impact of protein-changing status on the distribution of allele ages is statistically significant, but considerably smaller than the systematic differences introduced by demography and geography. Among the singleton protein-changing variants ascertained in the UK10k data, however, we find a larger difference with respect to variants that do not change proteins. The contrast with the 1KGP data is consistent with the younger, rarer, and more numerous variants of the UK10K data are subject to greater selective forces than the variants at 1% frequency in the 1KGP populations.

Our ANOVA did not reveal evidence that local adaptation contributes to the variance in ages of alleles at 1% frequency. If selectively important alleles were disproportionately prevented from migrating between populations or remaining in multiple populations, we would have observed the pool of shared protein-changing variants to be enriched for protein-changing variants without selective function. There would have been less differentiation between non-shared and shared, non-protein-changing variants, and there would have been a significant (negative) interaction term between private status and protein-changing status, which we did not observe. We also did not detect an interaction between populations and protein-changing status. If different populations were broadly experiencing greater or lesser amounts of directional selection on rare variants, or if there were substantial differences between populations in the ability of natural selection to remove harmful variants or raise the frequency of beneficial variants (such as due to variation in effective population size), we would expect to have seen a significant interaction between populations and protein-changing status. However, we did not; and while both of these phenomena may be taking place, they are not major drivers of the distribution of ages of variants found at 1% frequency, in contrast to the contributions of that demography, geographic spread, and global properties of natural selection.

These population genetic results are all presented as conditioning on alleles at 1% frequency that have been down-sampled to equal sizes. By considering only alleles of a particular frequency, we are able to draw meaningful comparisons and contrasts among populations. While the formula for the estimator itself is not a function of the frequency of the allele to which it is being applied, the distributions of ${\hat{t}}_{c}$ estimates will vary greatly as a function of the frequencies of the alleles being studied (see e.g. S15 Fig). Indeed, it is because of these very different age distributions that alleles of different frequencies can have very different histories, including the action of natural selection and the amount of movement among populations. Any future work that would make comparisons between populations or estimate demographic models based on ${\hat{t}}_{c}$ estimates will need to explicitly condition on allele frequencies.

With an estimate of an allele’s age, an investigator has an important new piece of information to bring to bear on the possible functional impact of a mutation. As shown here, for 1KGP data and even more strongly for the larger UK10K data, functional alleles are younger, and therefore, concomitantly, any allele that is discovered to be especially young, given its frequency in the sample, is a good candidate for having an effect of fitness. In this context, an increase in sample sizes will have multiple important effects. First, the rarest alleles in large samples will be rarer and younger, on average, than those found in smaller samples, and they will thereby be relatively enriched for more alleles of harmful effect and for alleles of more harmful effect (these are the alleles that would not reach those higher frequencies observable with smaller sample sizes). Second, the numbers of alleles in the rarest class rises with sample size, and thus so does the number of very rare alleles observed for a given gene. For example, the average number of autosomal singletons for the 26 1KGP populations (100 genomes) was 3,311,984, while the count for the UK10K data (7242 genomes) was approximately 6 times higher at 19,078,777. Based on those values, and assuming the number of SNPs is a function of the log of the sample size [5], a sample of 2*N* = 100,000 genomes would have 36 million singletons, and a sample of 1 million genomes would have 45 million singletons. The increasing density of very rare alleles, as sample sizes grow, opens the door to a kind of mapping of functional constraint across a gene that will become increasingly fine-grained. Third, as data sets get very large so grows the potential for studying the impact of selection on variation that has arisen at different times and it will become increasingly possible to assess whether the action of selection has been changing for different genes or regions of the genome.

The estimator can also be used to study forces that shape the overall distribution of allele ages across the genome, particularly demographic forces. As shown in Fig 5, the distributions of ages of singletons across 1KGP populations varies greatly, as do the differences between the ages of private and shared alleles. These distributions can be used in principle to develop models of demographic history, including in particular recent events that are only revealed by their impacts on the ages of very rare alleles [26].

## Methods

### Estimator development

#### Unknown sister(s)

Eq (1) assumes that the focal chromosome has a single known sister chromosome. However, even without knowing the sister(s), it is still possible to compare the focal chromosome to other chromosomes, and in each case measure the length of identity flanking the focal base. In the comparison with the (unknown) sister chromosome the distance will be a realization of a mutation process with parameter *μ*2*t*_*c*_, but for a non-sister the distance will be a realization of a process with parameter $\mu 2t_{c}^{*}$, where $t_{c}^{*}>t_{c}$ (because a sister chromosome is, by definition, the most closely related). It follows that for non-sisters the lengths of the regions identical to the focal chromosome flanking the focal base will be shorter on average than for the sister chromosome. For comparisons where $t_{c}^{*}\gg t_{c}$, the length of the tract of identity with the sister will be longer than the one with the more distant relative. The greater the difference between *t*_*c*_ and $t_{c}^{*}$, the more reliably the longest tract will come from an alignment with the sister chromosome. However, when $t_{c}^{*}≅t_{c}$, the longest tract may come from either alignment, but this is also the case where an estimate of $t_{c}^{*}$ based on the longest tract length is also a good estimate of *t*_*c*_. This suggests that it is not necessary to identify the sister(s), and that we can simply use the longest observed tract of identity. We call this quantity the maximum shared haplotype (*msh*). The *msh* can be measured by aligning the chromosome carrying the focal base with all other homologous chromosomes in the sample, and then measuring the distance between the focal base and the first non-identical base in each alignment. The largest such distance is the *msh*. The *msh* is also useful because it can be quickly measured, even for a large sample of chromosomes (see Extracting *msh* values from aligned sequence data).

#### The *msh* is primarily determined by events on branches A and S

The *msh* will have been caused by a mutation on one of the genealogy edges that has a location in the genealogy that is close to the edge associated with the focal base—indeed it will often be that very edge (A in Fig 1). However the *msh* value (i.e. the distance to this mutation) can only be revealed in comparison to other chromosomes, and this causes the *msh* to be a complex function of both the branch lengths and the topology of the genealogy.

We consider first the case of a genealogy that includes only multiple sister chromosomes. When the focal chromosome has multiple sisters, the density of the length of the tract of sequence identity between the focal chromosome and each individual sister is given by Eq (1), but the density of the longest tract from all of those sister alignments is not. However, no alignment between the focal chromosome and any of its sisters can extend past the first mutation arising on branch A or S, as mutations on either of these branches differentiate the focal chromosome from all of its sisters. Therefore, the distance *x*_*AS*_ from the focal base to the closest mutation having arisen on either branch A or S is an upper bound on the *msh*. However if every lineage in the sister clade has independently acquired a mutation closer to the focal base than *x*_*AS*_, then the *msh* will end at the most distant of these mutations, and will be shorter than *x*_*AS*_. This situation is diagrammed in Fig 1B and the general function for *msh* in the case of two sisters is shown in S4 Fig. It is most likely to occur in configurations where the focal base has only two sisters. In this case, the probability that *msh* is less than *x*_*AS*_ is the probability that two independent random variables drawn from an exponential distribution with a rate parameter *μ*(*t*_*c*_−*φ*) are both less than *x*_*AS*_, or

$$p({msh\neq x}_{AS})=\int_{0}^{\infty }\mu (t_{c}+\varphi )e^{-x_{AS}\mu (t_{c}+\varphi )}{\left[1-e^{-x_{AS}\mu (t_{c}-\varphi )}\right]}^{2}dx_{AS}=\frac{{\left(\varphi -t_{c}\right)}^{2}}{t_{c}(3t_{c}-\varphi )}$$

This probability quickly drops from a maximum of 1/3 when *φ* = 0 (when the time of the base of the sister clade is equal to *t*_*c*_), to 1/10 when *φ* = *t*_*c*_/2 and approaches zero as *φ* approaches *t*_*c*_.

It is also possible for the *msh* to be less than *x*_*AS*_ if the mutation causing the *msh* occurred outside of the clade that includes the focal base and its sister. In general this requires that both of the closest mutations on branches A or S were further away than the closest mutations on other branches that are near to the focal base in the genealogy, with the precise conditions becoming quite complex as sample size grows (S4 Fig).

Both sets of circumstances (i.e. *msh* < *x*_*AS*_ due to mutations on the sister clade, or due to mutations outside of the clade including the focal base and its sisters) have low probability as we show by simulation in S5 Fig. We therefore use the exponential density for *x*_*AS*_, which is a function of the lengths of only branches A and S (i.e. *t*_*c*_ + *φ*), to approximate the density of the *msh*,

$$p(msh|t_{c},\varphi ,\mu )≅{\mu (t_{c}+\varphi )e}^{-msh\mu (t_{c}+\varphi )}.$$ (2)

The length of the sister edge, *φ*, is not observable, however we show below the development of a coalescent approximation of the probability density, *p*(*φ*). Integrating over this density yields a likelihood function that includes the case when there is only one sister (*φ* = *t*_*c*_) and the range of possibilities when *φ* < *t*_*c*_:

$$p(msh|t_{c},\mu )≅\int_{0}^{t_{c}}p(\varphi ){\mu (t_{c}+\varphi )e}^{-msh\mu (t_{c}+\varphi )}d\varphi +p(\varphi =t_{c}){\mu 2t_{c}e}^{-msh\mu 2t_{c}}.$$ (3)

While the integration over *φ* does require a model of demographic history of the sample, in practice the choice of demographic model has little impact on the estimate (see below).

#### Including recombination

Recombination can be included in a manner similar to that of mutation, and we can envision a Poisson processes where each base has probabilities, *μ* and *ρ*, of mutating or recombining (respectively) in each generation. Looking out from the focal base, in comparisons between the focal chromosome and all other chromosomes, the tract over which there are no mutation or recombination events has a length that is exponentially distributed with a rate parameter (*μ* + *ρ*) [27, 28]. However, unlike mutation events that cause a clear difference between chromosomes, we cannot observe the base positions of recombination events directly.

In a system with recombination, the nearest recombination event to the focal base alters the genealogical relationship between the focal chromosome and its sisters. This is not an issue when a mutation occurs on branch A or S at a distance along the chromosome that is closer to the focal base than the first recombination event. However, when no mutation has occurred in the distance to the first recombination event, the chromosomes that were sisters up to the point of recombination, and that were acquiring differences from the focal chromosome at a rate of *μ*2*t*_*c*_ per site per generation, no longer share a common ancestor with the focal chromosome at *t*_*c*_. Instead, the recombination event will have introduced a random chromosome from the population, and for this the rate of appearance of differences with respect to the focal chromosome will be at the much higher rate of $\mu 2\overset{`}{t}$, where $\overset{`}{t}$ is a random draw from the distribution of times of common ancestry between two unrelated chromosomes in the sample. The effect of this higher rate of introduction of mutational differences means that the distance beyond the point of recombination to the first mutational difference will typically be short compared to the distance between the focal locus and the recombination event. We therefore approximate the density of *msh* for the case that includes recombination, by including recombination in the same way as we include mutation,

$$p(msh|t_{c},\mu ,\rho )≅\int_{0}^{t_{c}}p(\varphi ){(\mu +\rho )(t_{c}+\varphi )e}^{-msh(\mu +\rho )(t_{c}+\varphi )}d\varphi +p(\varphi =t_{c}){(\mu +\rho )2t_{c}e}^{-msh(\mu +\rho )2t_{c}}.$$ (4)

We assessed the difference between true *msh* values and expected *msh* value (by integration of Eq 4) using simulations with recombination. As shown in S6 Fig, the approximation introduces a modest bias, such that observed *msh* values tend to be longer on average than expected.

Eq (4) was developed by considering a tract of identify extending in one direction, either 5’ or 3’, from the focal base. In practice, investigators can record two *msh* values for every variant: *msh*_5_, extending from the focal base in the 5’ direction and *msh*_3_, in the 3’ direction. Each of these is ended by a different mutation or recombination event and therefore an independent observation (conditional on common values of *t*_*c*_ and *φ*). The joint likelihood of both *msh* values is the product of the probabilities of each one,

$$p({msh}_{5^{\prime }},{msh}_{3^{\prime }}|t_{c},\mu ,\rho )≅\int_{0}^{t_{c}}p(\varphi ){{\left(\left(\mu +\rho )(t_{c}+\varphi \right)\right)}^{2}e}^{-({msh}_{5^{\prime }}+{msh}_{3^{\prime }})(\mu +\rho )(t_{c}+\varphi )}d\varphi +p(\varphi =t_{c}){{\left((\mu +\rho )2t_{c}\right)}^{2}e}^{-({msh}_{5^{\prime }}+{msh}_{3^{\prime }})(\mu +\rho )2t_{c}}.$$ (5)

In practice, it is often the case that one of the *msh* values is not terminated by a difference but rather comes from a tract of identical sequence that continues to the end of a chromosome. If we denote this distance as *msh*_*eoc*_, we obtain a density comprised solely of the probability of no mutations or recombination events involving branches A or S within this distance,

$$p({msh}_{eoc}|t_{c},\varphi ,\mu ,\rho )≅e^{-{msh}_{eoc}(\mu +\rho )(t_{c}+\varphi )},$$

and a corresponding likelihood to be used when the *msh* in one direction is complete and the other is prematurely truncated:

$$p(msh,{msh}_{eoc}|t_{c},\mu ,\rho )≅\int_{0}^{t_{c}}p(\varphi ){{\left(\left(\mu +\rho )(t_{c}+\varphi \right)\right)}^{2}e}^{-(msh+{msh}_{eoc})(\mu +\rho )(t_{c}+\varphi )}d\varphi +p(\varphi =t_{c}){(\mu +\rho )2t_{c}e}^{-(msh+{msh}_{eoc})(\mu +\rho )2t_{c}}$$ (6)

Use of *msh* to estimate *t*_*c*_ is not limited to systems with uniform rates of recombination. For an arbitrary genetic map, the probability that no recombination events have occurred on branch A or S within a span of *c* Morgans is *e*^−*c*^, and the probability that the closest such recombination event occurs at a point *c* Morgans away from the focal base is *ρ*_*c*_*e*^−*c*^ where *ρ*_*c*_ is the per-base recombination rate at the site of the first recombination. We define *c*_5_, and *c*_3_, as the length in Morgans of the 5’ and 3’ *msh* tracts, *c*_*eoc*_ as the length in Morgans from a focal base to the end of the chromosome, and *ρ*_5_, and *ρ*_3_, as the per-base recombination rates at the ends of their respective msh tracts. The generalized versions of Eqs (5) and (6) are,

$$p(\chi |t_{c})≅\int_{0}^{t_{c}}p(\varphi ){\beta {\left(t_{c}+\varphi \right)}^{2}e}^{-\chi (t_{c}+\varphi )}d\varphi +p(\varphi =t_{c}){{\beta (2t_{c})}^{2}e}^{-\chi 2t_{c}}$$ (7)

And

$$p(\chi_{eoc}|t_{c})≅\int_{0}^{t_{c}}p(\varphi ){\beta_{eoc}(t_{c}+\varphi )e}^{-\chi_{eoc}(t_{c}+\varphi )}d\varphi +p(\varphi =t_{c}){\beta_{eoc}2t_{c}e}^{-\chi_{eoc}2t_{c}}$$ (8)

respectively, where, *χ* = *μ*(*msh*_5′_ + *msh*_3′_) + *c*_5′_, + *c*_3′_, *χ*_*eoc*_ = *μ*(*msh* + *msh*_*eoc*_) + *c* + *c*_*eoc*_, *β* = (*μ* + *ρ*_5′_)(*μ* + *ρ*_3′_), and *β*_*eoc*_ = *μ* + *ρ*.

#### The probability density of *φ* and final likelihood approximations

The final steps in developing a maximum likelihood estimator of *t*_*c*_ require an expression for *p*(*φ*). From Slatkin and Ranala [8], the number of branches ancestral to a sample of that existed *t* generations before a sample was taken can be approximated as

$$n(t)≅\frac{n}{1+\frac{n}{2}\tau (t)}$$

Where *n* is the sample size, *τ*(*t*) is the coalescent intensity integral, $\int_{0}^{t}\frac{1}{2N(\hat{t})}$, and $N(\hat{t})$ is the historical effective size of the population *t* generations before sampling. By substituting *t* = *t*_*c*_ − *φ* we reverse the time direction, and by taking the derivative with respect to *φ* we have an expression for the instantaneous rate of coalescence in the gene tree as time moves forward:

$$n^{\prime }(\varphi )={(\frac{n}{2N(t_{c}-\varphi )\tau (t_{c}-\varphi )+4})}^{2}.$$

The instantaneous rate of coalescence along one particular branch, our hazard function, *λ*(*φ*), is the rate for the tree divided by the number of branches:

$$\lambda (\varphi )=\frac{n^{\prime }(\varphi )}{n(\varphi )}.$$

The probability that the next coalescent event to happen on the branch occurs after *φ* generations is the product of the coalescent rate at generation *φ* and the probability of zero coalescent events in generations zero through:

$$f(\varphi )=\lambda (\varphi )e^{-\int_{0}^{\varphi }\lambda (\overset{´}{\varphi })d\overset{´}{\varphi }}.$$ (9)

The branch length *φ* has a maximum value of *t*_*c*_ generations, and if no coalescent event happens before that time the branch terminates at time zero instead of at a coalescent event. This event, with a point mass at *φ* = *t*_*c*_, corresponds to the case of the there being just one sister edge. In summary, the density for *φ* is

$$p(\varphi )=\{\begin{array}{cc} f(\varphi ), & for\;0\leq \varphi <t_{c}\\ 1-\int_{0}^{t_{c}}f(\varphi )d\varphi , & for\;\varphi =t_{c}\\ 0, & otherwise \end{array}.$$ (10)

For a model of a diploid population with a constant size, $\tau (t)=\frac{t}{2N}$ and $(t)=\frac{4nN}{4N+nt}$. Substituting *t*_*c*_ − *φ* for *t*, and carrying through Eqs (9) and (10), we obtain

$$p(\varphi )=\{\begin{array}{cc} \frac{n}{4N+nt_{c}}, & for\;0\leq \varphi <t_{c}\\ 1-\frac{nt_{c}}{4N+nt_{c}}, & for\;\varphi =t_{c}\\ 0, & otherwise \end{array}.$$ (11)

Substitution of Eq (11) for *p*(*φ*) in Eqs (7) and (8), followed by integration, provides the functions that were used for optimization and estimation of *t*_*c*_:

$$p(\chi |t_{c})≅\frac{e^{-\chi 2t_{c}}n(-2\beta (1+2t_{c}\chi (1+t_{c}\chi ))+e^{\chi t_{c}}\beta (2+t_{c}\chi (2+t_{c}\chi ))}{(4N_{0}+nt_{c})\chi^{3}}+\frac{16e^{-\chi 2t_{c}}N_{0}{t_{c}}^{2}\beta }{4N_{0}+nt_{c}}$$ (12)

And

$$p(\chi_{eoc}|t_{c})≅\frac{e^{-\chi 2t_{c}}n\beta_{eoc}(-1-2t_{c}\chi +e^{\chi_{eoc}t_{c}}(1+t_{c}\chi )}{(4N_{0}+nt_{c}){\chi_{eoc}}^{2}}+\frac{8e^{-\chi_{eoc}2t_{c}}N_{0}t_{c}\beta_{eoc}}{4N_{0}+nt_{c}}$$ (13)

for the cases when the *msh* can be measured in both directions, and when direction is prematurely truncated by the end of the chromosome, respectively. We observed that Eqs (12) and (13), and estimates obtained using them, are weak functions of *N*_0_. More generally we found that using either *N* = 10^4^ or *N* = 10^5^ has very little effect on estimates of *t*_*c*_, and that alternative expressions, found by integration over *f*(*φ*) using a demographic model of recent strong exponential growth, gave estimates of *t*_*c*_ that were nearly identical to those obtained using Eqs (12) and (13) which assumed a constant size of *N* (S5 Table, S7 Fig).

#### *t*_*c*_ estimation and singleton phase estimation

The approximate likelihoods given above can be used to estimate the first coalescent time at any base position, for any chromosome in a sample of chromosomes from a population. In addition, these expressions can be easily adapted to the case when a focal base on a focal chromosome carries a derived allele at a SNP. Consider the case of a SNP in which the derived allele occurs just once in the sample (a singleton). If this singleton is the focal base, then there must have been at least one mutation on edge A and the time of that mutation could not have been greater than *t*_*c*_. With a constant mutation rate per base of *μ*, the probability of one or more mutations on this edge is $\left(1-e^{-\mu t_{c}}\right)$. Thus we adapt the likelihoods used for estimating *t*_*c*_ to the case of a single derived allele at the focal base, simply by including this probability in the likelihood.

For variants found more than once in a sample, we record *χ* for each of the *k* copies of the rare allele, which is the longest tracts of identity it shares with a chromosome that does not carry the rare allele. The product, $\prod_{i=1}^{k}p(\chi_{i}t_{c})$, gives a composite likelihood that can be maximized to estimate *t*_*c*_.

The property by which the probability of a branch harboring a mutation increases with the length of the branch also provides information about the correct haplotype phasing of singleton variants. Consider the position of the focal base on the two chromosomes of a diploid individual that is heterozygous for a singleton variant at that base position. One of the chromosomes carries a mutation that occurred at the focal base position between *t*_*c*_ and the time of sampling, whereas no mutation has occurred on the individual’s other chromosome at this base position since its first coalescent time (a time point we identify as $t_{c}^{*}$). As the probability of a mutation on a genealogical branch is proportional to the length of the branch, assigning the singleton variant to the haplotype with the larger estimated *t*_*c*_ should correctly phase the variant with a probability equal to $t_{c}/(t_{c}+t_{c}^{*})$. When the difference between *t*_*c*_ and $t_{c}^{*}$ is large, the probability of correctly phasing the singleton variant is high. When $t_{c}^{*}≅t_{c}$, the probability of correctly phasing the singleton variant drops towards 0.5. In this case, however, the estimate of *t*_*c*_ is approximately correct even when phase is incorrectly assigned.

We developed software to estimate *t*_*c*_ values for every genetic variant in a haplotype-phased VCF file given a demographic history, genomic mutation rate, and genetic map. We adapted the positional Burrows–Wheeler transform (PBWT) algorithm of Durbin [23] to extract the lengths and locations of *msh*_5′_ and *msh*_3′_ tracts for each variant. These *msh*_5′_ and *msh*_3′_ tracts are converted into *χ* values, and *t*_*c*_ is estimated by maximizing Eqs (12) and (13) using the Brent optimization routine implemented in the Python module scipy.optimize. The PBWT algorithm can be rapidly applied and has linear time complexity with sample size, allowing for rapid calculation of large numbers of ${\hat{t}}_{c}$ values even for very large numbers of chromosomes.

#### Extracting *msh* values from aligned sequence data

We extract all *msh* values from a VCF file using prefix arrays generated by the positional Burrows-Wheeler transform. Following algorithm 2 in Durbin [29], we create matrices *a* and *d* from the sequence alignment. Both matrices have a row for each chromosome and a column for each position in the VCF file. Each column *k* of matrix *a* orders the chromosomes to give the longest matching alignment preceding site *k* between consecutive chromosomes. Matrix *d* records the lengths of all the preceding alignment matches when the chromosomes are ordered as in matrix *a*.

For a singleton variant located at focal base *k* of a focal chromosome, the start of the *msh*_5′_ tract is read by identifying the position *j* of the focal chromosome in the *k*-th column of matrix *a* and taking the minimum of the values of the *j*-th and (*j* + 1)-th values of the *k*-th column of matrix *d*. By reversing the VCF file and repeating algorithm 2 we create a second set of *a* and *d* matrices with which we derive *msh*_3′_ tracts. Using the physical locations of variants in the VCF file and a human reference genetic map, the start and end points of the *msh* tracts are transformed into values of *χ* necessary for estimation of *t*_*c*_.

Matrix *d* is also sufficient to ascertain the length of the *msh* between any pair of chromosomes at every locus in the genome. For the row of matrix *d* corresponding to a position of interest, the maximum entry found between two chromosomes indicates the position of the most recent non-shared site between them. From this observation, for a variant observed at greater than singleton frequency, we calculate an *msh* for each instance of the variant as the longest *msh* found between the chromosome carrying the variant and any chromosome not carrying the variant.

### Data

We applied the estimator of *t*_*c*_ to low frequency alleles in data from 26 human populations, grouped into five super-populations, from the 1000 Genomes Project (1KGP) data set [15]. We sub-sampled each population to achieve a uniform sample size of *N* = 50 diploid individuals. Chromosomes were taken as phased by the 1000 Genomes Consortium using SHAPEIT [30] with the exception that we re-phased singleton variants in each population based on relative ${\hat{t}}_{c}$ values. To do this, for each heterozygous singleton, we masked all other singleton variants, calculated ${\hat{t}}_{c}$ for both possible phases, and assigned the variant to the chromosome producing the larger ${\hat{t}}_{c}.$ *t*_*c*_ values were estimated separately for each population, not collectively as a single pooled sample.

We also estimated *t*_*c*_ for singleton variants in the mapping sample of 3,621 individuals (7,242 genomes) from the UK10k data set that has been filtered by the UK10k consortium to remove close relatives and individuals of recent non-European ancestry [16]. These include genomes from the ALSPAC cohort, which focused on the Avon region, and the TWINSUK cohort which includes samples from across the UK. Haplotype phase for variants found two or more times was inferred by the UK10k consortium using SHAPEIT. Haplotype phase for singleton variants was determined as for the 1KGP data.

For all data we masked TpG/CpG transitions to minimize mutation rate variability. We compared results found using genetic maps based on both linkage disequilibrium [26] and pedigrees [31], and found very little difference in *t*_*c*_ estimates (S8 Fig). Results presented here used the pedigree-based map. We assume the (non-CpG) mutation rate is constant and equal to 1 × 10^−8^ per base per generation [32–34]. We assume a demographic history of constant population size of *N* = 1 × 10^4^.

### Simulations

Data were simulated using the msprime program [35] under three demographic models: a constant population of size *N* = 1 × 10^4^; a population with recent exponential growth that started from an ancestral population size of *N*_*a*_ = 1 × 10^4^ to a population size of *N* = 5 × 10^5^ over the last 200 generations prior to sampling; and a bottleneck model in which samples were drawn from a ‘European’ population included in an out-of-Africa model with exponential growth and migration using the parameter estimates of Gutenkunst et al., [24]. We simulated samples of 1000 10-Megabases chromosomes from which we sub-sampled populations of 100 chromosomes for comparison with 1KGP data. We used a fixed mutation rate of *μ* = 1 × 10^−8^ per base per generation, as is typical for human populations [32–34]. For recombination rate we used a value of *ρ* = 1 × 10^−8^ that is typical of the mean rate per base pair in human populations [26, 31], as well as both a lower recombination rate (*ρ* = 1 × 10^−9^) and a higher recombination rate (*ρ* = 1 × 10^−7^) to assess performance when a different proportion of *msh* tracts end in a variant that has been introduced to the focal chromosome or a sister chromosome by recombination, and not directly by mutation.

We assess error as the root mean squared error of *log*_10_ transformed *t*_*c*_ values, and bias as the average signed error of the *log*_10_ transformed *t*_*c*_ values. To assess the impact of phase uncertainty we randomly paired chromosomes and phased the data using SHAPEIT with default parameters. Singletons were phased as for the 1KGP and UK10K data.

### Data analysis

We used SNPEff [36] to identify variants annotated as missense, stop gained, stop lost, start lost, splice acceptor, or splice donor as ‘protein changing’. For singleton variants in UK10K populations, a variant is labeled as ‘private’ when it is found in only one population, and ‘shared’ when it is also found in other populations (at any frequency).

Singleton variant ages in the 1KGP data were analyzed with a type-II ANOVA that examined the effect of three variables, including: (1) the population in which the variant was observed; (2) whether or not a variant is private to a single populations; and (3) whether or not a variant effects a protein sequence. The model has the form *log*_10_(*t*_*c*_) ~ *Intercept* + *PC* + *Private* + *Population* + (*PC* × *Population*) + (*Private* × *Population*) + (*PC* × *Private*), where *PC* is a categorical variable indicating protein-changing status, *Private* is a categorical variable indicating the restriction of a variant to a single population, and *Population* is a categorical variable indicating the population in which a variant is observed.

### Ethics statement

The only human data used in this study are genome sequences that are publicly available. Two sources of human data were used. The 1000 Genomes data were downloaded from the data server at http://www.internationalgenome.org/data. The UK10K data are made available to researchers as described (https://www.uk10k.org/data_access.html) and were made available to Dr. Hey following completion of the UK10K Project Data Access Agreement (signed 14 Oct 2014). These data included dataset IDS EGAS00001000090 (UK10K COHORT ALSPAC) and EGAS00001000108 (UK10K COHORT TWINSUK). These data include no information such that the original subjects could be identified. Because all of the data is publicly available, and because none of the data can be used to identify the original subjects, the study falls under NIH Human Subjects Research Exemption 4. For these same reasons the research was determined to not involve Human Subjects by Temple University IRB.

## Web resources

The program, entitled “runtc”, that implements the estimator for VSF files is available at https://github.com/jaredgk/msh-python/tree/master/msh_est.

The simulation results are available at https://bio.cst.temple.edu/~hey/nolinks/Platt_etal_SimulationsAndAnalyses.zip.

## Supporting information

S1 Table Performance of *t*_*c*_ estimator under simulated models. Columns are shown for Error (root squared mean error of *log*_10_(*t*_*c*_)), Error *t*_*c*_ (rmse of *t*_*c*_), bias (mean signed error of *log*_10_(*t*_*c*_)), and *r* (Pearson’s correlation) for the true and estimated values of *log*_10_(*t*_*c*_). Results are shown for sample sizes of 100 and 1000 chromosomes drawn from populations with a constant size of *N* = 1 × 10^4^ (constant), one with an historical size of *N* = 1 × 10^4^ and exponential growth over the last 200 generations to a size of *N* = 5 × 10^5^ at the time of sampling (recent growth), or a complex model of European history derived from Gutenkunst *et al*. [24] including a large ancestral African population with a population bottleneck, migration to and from diverging African and Asian populations, and recent exponential growth (Out of Africa). Each model is presented at two constant recombination rates, and evaluated with both perfectly phased haplotypes taken from simulation (known) or haplotypes statistically phased from simulated diploid genotypes (inferred). The estimator is evaluated for all alleles in each sample, as well as subsets of alleles of increasing rarity.
(DOCX)

S2 Table Estimator performance with high recombination. Results are shown for constant and growing populations for high recombination (see S1 Table).
(DOCX)

S3 Table Mean of *log*_10_ transformed singleton *t*_*c*_ values, before and after transforming back to natural scale for each 1KGP population. (DOCX)

S4 Table Timings for determination of *msh* values and *t*_*c*_ estimates for singleton variants in the UK10K chromosome 22 data by sample size and number of variants. (DOCX)

S5 TableDifferences in ${\hat{t}}_{c}$ values across changes in demographic assumptions when determining the density of the edge leading to the sister clade, (*φ*|*t*_*c*_).Data were simulated to generate sample of 100 and 1000 chromosomes under each of two recombination rates (1 × 10−9 and 1 × 10−8), and under each of two demographic models: one with a constant size of *N* = 1 × 104; and one with an historical size of *N* = 1 × 104 followed by exponential growth over the last 200 generations to a final size of *N* = 5 × 105. Two versions of the tc estimator were applied to all alleles that occurred in each of the 8 simulations, one that assumed a constant *N* = 1 × 104 and a second that assumed a constant *N* = 5 × 105. Results are the average difference in ${log}_{10}({\hat{t}}_{c})$ values and Pearson’s r statistic between the ${log}_{10}({\hat{t}}_{c})$ values.(DOCX)

S6 Table Table of coefficients for each categorical variable in ANOVA of *log*_10_(*t*_*c*_) values of singleton variants in each population of the 1KGP data. (DOCX)

S1 Fig Comparisons of *msh*-based *t*_*c*_ estimator with idealized frequency-based estimator. Using the data from the simulations shown in Fig 2, these boxplots show the increase in precision in estimating *t*_*c*_ from *msh* compared to the best possible estimator operating on variant frequency. Cells a-c represent samples of *n* = 1000. Cells d-e represent samples of *n* = 100. a and d include all variants of frequency ≤ 10%, b and e represent variants of frequency ≤ 1%, and c is only variants found at 0.1% frequency.
(TIF)

S2 Fig${\hat{t}}_{c}$ values for 82563 singletons male X chromosomes from the UK10K panel.The X axis shows values for a set of original X chromosomes, and the Y chromosome shows values for the same data after randomization to simulate 50 (diploid) pairs of unphased chromosomes, followed by application of phasing software and singleton phasing as described in the manuscript. To generate rephrased data, 100 chromosomes were randomly paired, and heterozygous positions were randomized to simulate a diploid, short-read, assembly, and then phased using SHAPEIT [37]. We used a mutation rate of 10^−8^ per base pair and the deCODE genetic map [31, 38].(TIF)

S3 Fig Loci with multiple independently derived minor alleles. Shown is the case in which a locus harbors both a singleton variant and a false-positive SNP call. 1000 doubleton variants are generated by randomly selecting a singleton SNP in the UK10K sample, and then selecting an additional chromosome at random and assigning to it the derived allele. We compare the original singleton *t*_*c*_ estimate with the *t*_*c*_ estimate of the artificially created doubleton variant. The addition of a derived allele that is identical by state but not identical by descent does not produce radically deviant estimates. True double mutations are expected to behave in a similar manner, though are more likely to be found on longer branches than randomly chosen branches used for this figure.
(TIF)

S4 Fig Gene trees showing a chromosome at the focal base (white), sister chromosomes (blue) and other sampled chromosomes (black). Panels A, B and C show three different sample sizes. Within each panel, every edge has a value in italics (*A*, *B*, *C*, *D* etc) that is the distance from the focal base to the closest mutation to that base that occurred on that edge. Below each figure is given the maximum shared haplotype (*msh*) value as a function of the distances to the mutations on each edge.
(TIF)

S5 FigMaximum shared haplotype (*msh*) and the distance to the first mutation on either branch A or S.*x*_*AS*_ are plotted against the corresponding *msh* values as determined in a set of simulated data. Coalescent simulations (10^4^ independent simulations) were conducted for a sample of 100 chromosomes drawn from a constant-sized diploid population of *N* = 10^6^, with a per base mutation rate of 2 × 10^−8^, and no recombination using msprime [35]. For each simulated data set we measured *msh* of a singleton variant and the longest maximum shared haplotype determined by considering only events on the external branch immediately ancestral to the singleton variant and its first sister branch (approximate *msh*).(TIF)

S6 Fig *msh* values as a function of *t*_*c*_. Black dots represent observed *msh*, values from singleton variants from *n* = 100 chromosomes sampled from a simulated constant-sized population of *N* = 1 × 10^4^ individuals with per-base mutation and recombination rates of *μ* = *ρ* = 1 × 10^−8^ as a function of their true *t*_*c*_ values. The red line is the expected value of a one-direction *msh* tract as a function of *t*_*c*_, given the same parameter values, obtained by integration of Eq 4 (slope: -0.961, intercept: 7.353). The blue line is the linear regression of *log*_10_(*msh*) on *log*_10_(*t*_*c*_) (slope: -0.703, intercept: 6.858).
(TIF)

S7 Fig Values of *t*_*c*_ estimated under two assumptions of demographic history. Variants from 100 chromosomes simulated from a population with an historic size of *N* = 1 × 10^4^ and exponential growth over the last 200 generations to a size of *N* = 5 × 10^5^ at the time of sampling (recent growth) have *t*_*c*_ estimated under extreme assumptions of a constant demography of *N* = 1 × 10^4^ and a constant demography of *N* = 5 × 10^5^.
(TIF)

S8 FigComparison of results with different genetic maps.${Log}_{10}({\hat{t}}_{c})$ values for 88,651 low frequency UK10K alleles from chromosome 22 with counts between 2 and 10, inclusive. Estimates were generated using the HapMap [26] genetic map, which has a total length of 80 centimorgans, and the deCODE map [31], which has a total length of 55 centimorgans. Pearson’s correlation coefficient is 0.929.(TIF)

S9 FigObserved frequencies of ${log}_{10}({\hat{t}}_{c})$ values for singleton alleles in African populations.Distributions are shown for private alleles and alleles that are also found in other populations, and for both.(TIF)

S10 FigObserved frequencies of ${log}_{10}({\hat{t}}_{c})$ values for singleton alleles in European populations.Distributions are shown for private alleles and alleles that are also found in other populations, and for both.(TIF)

S11 FigObserved frequencies of ${log}_{10}({\hat{t}}_{c})$ values for singleton alleles in East Asian populations.Distributions are shown for private alleles and alleles that are also found in other populations, and for both.(TIF)

S12 FigObserved frequencies of ${log}_{10}({\hat{t}}_{c})$ values for singleton alleles in South Asian populations.Distributions are shown for private alleles and alleles that are also found in other populations, and for both.(TIF)

S13 FigObserved frequencies of ${log}_{10}({\hat{t}}_{c})$ values for singleton alleles in admixed American populations.Distributions are shown for private alleles and alleles that are also found in other populations, and for both.(TIF)

S14 FigObserved frequencies of ${log}_{10}({\hat{t}}_{c})$ values for singleton alleles in an Out-of-African simulation.Following the parameter estimates of Gutenkunst et al., [24], 100 chromosomes of length 109 bases for each population were simulated using SCRM [39], with per base mutation rates of 1e-8 for both mutation and recombination. Distributions are shown for Europe (A), Asia (B) and Africa (C) for private alleles and alleles that are also found in other populations, and for both.(TIF)

S15 FigObserved frequencies of ${log}_{10}({\hat{t}}_{c})$ values for the CEU population of the 1KG data from alleles observed once, twice and five times.(TIF)
