# Supplementary material for: An estimator of first coalescent time reveals selection on young variants and large heterogeneity in rare allele ages among human populations
Source: PLoS Genet. 2019 Aug 19;15(8):e1008340. doi: 10.1371/journal.pgen.1008340 (PMC6715256; doi:10.1371/journal.pgen.1008340)
Supplement: S1 Table — Columns are shown for Error (root squared mean error of log10(tc)), Error tc (rmse of tc), bias (mean signed error of log10(tc)), and r (Pearson’s correlation) for the true and estimated values of log10(tc). Results are shown for sample sizes of 100 and 1000 chromosomes drawn from populations with a constant size of N = 1 × 10^4^ (constant), one with an historical size of N = 1 × 10^4^ and exponential growth over the last 200 generations to a size of N = 5 × 10^5^ at the time of sampling (recent growth), or a complex model of European history derived from Gutenkunst et al. [24] including a large ancestral African population with a population bottleneck, migration to and from diverging African and Asian populations, and recent exponential growth (Out of Africa). Each model is presented at two constant recombination rates, and evaluated with both perfectly phased haplotypes taken from simulation (known) or haplotypes statistically phased from simulated diploid genotypes (inferred). The estimator is evaluated for all alleles in each sample, as well as subsets of alleles of increasing rarity. (DOCX) [file pgen.1008340.s001.docx]

| sample size | demography | recombination  rate | phasing | allele frequencies | Error | Error  $t_{c}$ | Bias | $r$ |
| --- | --- | --- | --- | --- | --- | --- | --- | --- |
| 100 | constant | $1\times{10}^{-9}$ | known | all | 0.4 | 2.2x10⁵ | -0.22 | 0.92 |
|  |  |  |  | $\leq$10% | 0.38 | 7.7x10⁴ | -0.22 | 0.9 |
|  |  |  |  | 1% | 0.41 | 7.0x10⁴ | -0.24 | 0.88 |
|  |  |  | inferred | all | 0.43 | 2.2x10⁵ | -0.24 | 0.89 |
|  |  |  |  | $\leq$10% | 0.45 | 6.6x10⁴ | -0.25 | 0.84 |
|  |  |  |  | 1% | 0.55 | 3.1x10⁴ | -0.35 | 0.76 |
|  |  | $1\times{10}^{-8}$ | known | all | 0.44 | 9.3x10⁴ | 0.09 | 0.82 |
|  |  |  |  | $\leq$10% | 0.38 | 3.8x10⁴ | -0.03 | 0.82 |
|  |  |  |  | 1% | 0.37 | 2.2x10⁴ | -0.13 | 0.84 |
|  |  |  | inferred | all | 0.47 | 9.3x10⁴ | 0.08 | 0.79 |
|  |  |  |  | $\leq$10% | 0.43 | 3.7x10⁴ | -0.05 | 0.77 |
|  |  |  |  | 1% | 0.49 | 2.0x10⁴ | -0.21 | 0.73 |
|  | recent growth | $1\times{10}^{-9}$ | known | all | 0.39 | 2.8x10⁵ | -0.22 | 0.92 |
|  |  |  |  | $\leq$10% | 0.38 | 1.9x10⁵ | -0.22 | 0.89 |
|  |  |  |  | 1% | 0.4 | 3.8x10⁴ | -0.24 | 0.85 |
|  |  |  | inferred | all | 0.45 | 2.8x10⁵ | -0.25 | 0.88 |
|  |  |  |  | $\leq$10% | 0.47 | 1.9x10⁵ | -0.27 | 0.83 |
|  |  |  |  | 1% | 0.57 | 3.4x10⁴ | -0.38 | 0.71 |
|  |  | $1\times{10}^{-8}$ | known | all | 0.44 | 1.3x10⁵ | 0.09 | 0.82 |
|  |  |  |  | $\leq$10% | 0.38 | 5.4x10⁴ | -0.03 | 0.82 |
|  |  |  |  | 1% | 0.37 | 1.8x10⁴ | -0.13 | 0.8 |
|  |  |  | inferred | all | 0.48 | 1.3x10⁵ | 0.07 | 0.78 |
|  |  |  |  | $\leq$10% | 0.44 | 5.4x10⁴ | -0.07 | 0.74 |
|  |  |  |  | 1% | 0.5 | 1.6x10⁴ | -0.25 | 0.66 |
|  | Out of Africa | $1\times{10}^{-9}$ | known | all | 0.41 | 2.3x10⁵ | -0.23 | 0.95 |
|  | (European) |  |  | $\leq$10% | 0.39 | 1.7x10⁵ | -0.20 | 0.94 |
|  |  |  |  | 1% | 0.41 | 1.7x10⁵ | -0.22 | 0.94 |
|  |  |  | inferred | all | 0.49 | 2.0x10⁵ | -0.25 | 0.91 |
|  |  |  |  | $\leq$10% | 0.50 | 1.6x10⁵ | -0.22 | 0.89 |
|  |  |  |  | 1% | 0.55 | 1.7x10⁵ | -0.35 | 0.89 |
|  |  | $1\times{10}^{-8}$ | known | all | 0.38 | 1.4x10⁵ | 0.04 | 0.93 |
|  |  |  |  | $\leq$10% | 0.37 | 1.1x10⁵ | 0.04 | 0.92 |
|  |  |  |  | 1% | 0.36 | 9.4x10⁴ | 0.01 | 0.93 |
|  |  |  | inferred | all | 0.44 | 1.4x10⁵ | 0.01 | 0.90 |
|  |  |  |  | $\leq$10% | 0.44 | 1.1x10⁵ | 0.00 | 0.88 |
|  |  |  |  | 1% | 0.44 | 9.7x10⁴ | -0.09 | 0.88 |
| 1000 | constant | $1\times{10}^{-9}$ | known | all | 0.39 | 1.5x10⁵ | -0.2 | 0.95 |
|  |  |  |  | $\leq$10% | 0.37 | 7.9x10⁴ | -0.21 | 0.94 |
|  |  |  |  | $\leq$1% | 0.38 | 4.9x10⁴ | -0.23 | 0.91 |
|  |  |  |  | 0.1% | 0.4 | 9.1x10³ | -0.25 | 0.88 |
|  |  |  | inferred | all | 0.41 | 1.5x10⁵ | -0.21 | 0.94 |
|  |  |  |  | $\leq$10% | 0.41 | 7.8x10⁴ | -0.23 | 0.92 |
|  |  |  |  | $\leq$1% | 0.44 | 4.9x10⁴ | -0.26 | 0.88 |
|  |  |  |  | 0.1% | 0.54 | 7.9x10³ | -0.36 | 0.78 |
|  |  | $1\times{10}^{-8}$ | known | all | 0.5 | 8.9x10⁴ | 0.1 | 0.88 |
|  |  |  |  | $\leq$10% | 0.4 | 5.0x10⁴ | -0.04 | 0.88 |
|  |  |  |  | $\leq$1% | 0.37 | 2.0x10⁴ | -0.16 | 0.84 |
|  |  |  |  | 0.1% | 0.39 | 7.2x10³ | -0.22 | 0.88 |
|  |  |  | inferred | all | 0.52 | 8.9x10⁴ | 0.09 | 0.86 |
|  |  |  |  | $\leq$10% | 0.44 | 5.0x10⁴ | -0.05 | 0.86 |
|  |  |  |  | $\leq$1% | 0.43 | 2.0x10⁴ | -0.19 | 0.84 |
|  |  |  |  | 0.1% | 0.52 | 6.7x10³ | -0.32 | 0.79 |
|  | recent growth | $1\times{10}^{-9}$ | known | all | 0.38 | 1.2x10⁵ | -0.19 | 0.94 |
|  |  |  |  | $\leq$10% | 0.36 | 6.0x10⁴ | -0.2 | 0.91 |
|  |  |  |  | $\leq$1% | 0.37 | 1.9x10⁴ | -0.22 | 0.82 |
|  |  |  |  | 0.1% | 0.39 | 4.9x10³ | -0.23 | 0.64 |
|  |  |  | inferred | all | 0.45 | 1.2x10⁵ | -0.24 | 0.91 |
|  |  |  |  | $\leq$10% | 0.46 | 5.9x10⁴ | -0.27 | 0.86 |
|  |  |  |  | $\leq$1% | 0.49 | 1.9x10⁴ | -0.31 | 0.69 |
|  |  |  |  | 0.1% | 0.56 | 4.0x10³ | -0.4 | 0.45 |
|  |  | $1\times{10}^{-8}$ | known | all | 0.48 | 6.5x10⁴ | 0.07 | 0.86 |
|  |  |  |  | $\leq$10% | 0.38 | 2.5x10⁴ | -0.07 | 0.84 |
|  |  |  |  | $\leq$1% | 0.34 | 1.1x10⁴ | -0.16 | 0.77 |
|  |  |  |  | 0.1% | 0.36 | 6.0x10³ | -0.2 | 0.62 |
|  |  |  | inferred | all | 0.53 | 6.5x10⁴ | 0.02 | 0.82 |
|  |  |  |  | $\leq$10% | 0.45 | 2.4x10⁴ | -0.13 | 0.77 |
|  |  |  |  | $\leq$1% | 0.45 | 1.1x10⁴ | -0.25 | 0.64 |
|  |  |  |  | 0.1% | 0.51 | 6.0x10³ | -0.36 | 0.44 |
|  | Out of Africa | $1\times{10}^{-9}$ | known | all | 0.40 | 2.5x10⁵ | -0.23 | 0.96 |
|  | (European) |  |  | $\leq$10% | 0.38 | 1.9x10⁵ | -0.22 | 0.96 |
|  |  |  |  | $\leq$1% | 0.39 | 1.7x10⁵ | -0.22 | 0.96 |
|  |  |  |  | 0.1% | 0.39 | 1.3x10⁵ | -0.23 | 0.92 |
|  |  |  | inferred | all | 0.45 | 2.1x10⁵ | -0.21 | 0.94 |
|  |  |  |  | $\leq$10% | 0.45 | 1.7x10⁵ | -0.22 | 0.93 |
|  |  |  |  | $\leq$1% | 0.47 | 1.5x10⁵ | -0.24 | 0.92 |
|  |  |  |  | 0.1% | 0.55 | 1.3x10⁵ | -0.36 | 0.85 |
|  |  | $1\times{10}^{-8}$ | known | all | 0.44 | 1.2x10⁵ | 0.05 | 0.92 |
|  |  |  |  | $\leq$10% | 0.39 | 7.6x10⁴ | 0.01 | 0.92 |
|  |  |  |  | $\leq$1% | 0.34 | 5.1x10⁴ | -0.05 | 0.92 |
|  |  |  |  | 0.1% | 0.35 | 4.9x10⁴ | -0.11 | 0.91 |
|  |  |  | inferred | all | 0.52 | 1.1x10⁵ | 0.06 | 0.88 |
|  |  |  |  | $\leq$10% | 0.47 | 6.8x10⁴ | 0.00 | 0.88 |
|  |  |  |  | $\leq$1% | 0.45 | 4.7x10⁴ | -0.07 | 0.86 |
|  |  |  |  | 0.1% | 0.49 | 4.9x10⁴ | -0.25 | 0.84 |
