# Supplementary material for: An estimator of first coalescent time reveals selection on young variants and large heterogeneity in rare allele ages among human populations
Source: PLoS Genet. 2019 Aug 19;15(8):e1008340. doi: 10.1371/journal.pgen.1008340 (PMC6715256; doi:10.1371/journal.pgen.1008340)
Supplement: S2 Table — Results are shown for constant and growing populations for high recombination (see S1 Table). (DOCX) [file pgen.1008340.s002.docx]

| sample size | demography | recombination  rate | phasing | allele frequencies | Error | Error  $t_{c}$ | Bias | $r$ |
| --- | --- | --- | --- | --- | --- | --- | --- | --- |
| 100 | constant | $1\times{10}^{-7}$ | known | all | 1.07 | 3.4x10⁴ | 0.88 | 0.56 |
|  |  |  |  | $\leq$10% | 0.80 | 1.7x10⁴ | 0.57 | 0.53 |
|  |  |  |  | 1% | 0.57 | 9.2x10³ | 0.28 | 0.62 |
|  |  |  | inferred | all | 1.08 | 3.4x10⁴ | 0.87 | 0.50 |
|  |  |  |  | $\leq$10% | 0.81 | 1.7x10⁴ | 0.55 | 0.43 |
|  |  |  |  | 1% | 0.59 | 9.3x10³ | 0.21 | 0.47 |
|  | recent growth | $1\times{10}^{-7}$ | known | all | 1.05 | 3.4x10⁴ | 0.85 | 0.57 |
|  |  |  |  | $\leq$10% | 0.76 | 2.1x10⁴ | 0.54 | 0.52 |
|  |  |  |  | 1% | 0.50 | 7.4x10³ | 0.24 | 0.56 |
|  |  |  | inferred | all | 1.06 | 3.4x10⁴ | 0.83 | 0.49 |
|  |  |  |  | $\leq$10% | 0.78 | 2.1x10⁴ | 0.51 | 0.39 |
|  |  |  |  | 1% | 0.52 | 7.5x10³ | 0.15 | 0.40 |
| 1000 | constant | $1\times{10}^{-7}$ | known | all | 1.07 | 2.8x10⁴ | 0.76 | 0.70 |
|  |  |  |  | $\leq$10% | 0.76 | 1.2x10⁴ | 0.42 | 0.70 |
|  |  |  |  | $\leq$1% | 0.47 | 5.5x10³ | 0.09 | 0.77 |
|  |  |  |  | 0.1% | 0.38 | 2.7x10³ | -0.08 | 0.81 |
|  |  |  | inferred | all | 1.08 | 2.8x10⁴ | 0.75 | 0.67 |
|  |  |  |  | $\leq$10% | 0.78 | 1.2x10⁴ | 0.41 | 0.65 |
|  |  |  |  | $\leq$1% | 0.52 | 5.5x10³ | 0.07 | 0.69 |
|  |  |  |  | 0.1% | 0.48 | 2.7x10³ | -0.18 | 0.71 |
|  | recent growth | $1\times{10}^{-7}$ | known | all | 0.92 | 2.5x10⁴ | 0.55 | 0.67 |
|  |  |  |  | $\leq$10% | 0.62 | 1.1x10⁴ | 0.27 | 0.60 |
|  |  |  |  | $\leq$1% | 0.36 | 4.6x10³ | 0.04 | 0.59 |
|  |  |  |  | 0.1% | 0.29 | 1.6x10³ | -0.07 | 0.51 |
|  |  |  | inferred | all | 0.94 | 2.5x10⁴ | 0.50 | 0.57 |
|  |  |  |  | $\leq$10% | 0.65 | 1.1x10⁴ | 0.21 | 0.46 |
|  |  |  |  | $\leq$1% | 0.42 | 4.6x10³ | -0.04 | 0.40 |
|  |  |  |  | 0.1% | 0.37 | 1.7x10³ | -0.21 | 0.32 |
