# Supplementary material for: An estimator of first coalescent time reveals selection on young variants and large heterogeneity in rare allele ages among human populations
Source: PLoS Genet. 2019 Aug 19;15(8):e1008340. doi: 10.1371/journal.pgen.1008340 (PMC6715256; doi:10.1371/journal.pgen.1008340)
Supplement: S3 Table — (DOCX) [file pgen.1008340.s003.docx]

| Super-population | Population | **mean** ${log}_{10}(t_{c})$ | | natural scale (generations) |
| --- | --- | --- | --- | --- |
| African | ACB -- African Caribbeans | 3.83 | 6,761 | |
|  | ASW -- Americans of African Ancestry | 3.81 | 6,457 | |
|  | ESN -- Esan | 3.84 | 6,918 | |
|  | GWD -- Gambian | 3.8 | 6,310 | |
|  | LWK -- Luhya | 3.86 | 7,244 | |
|  | MSL -- Mende | 3.81 | 6,457 | |
|  | YRI -- Yoruban | 3.83 | 6,761 | |
| Admixed American | CLM -- Colombian | 3.84 | 6,918 | |
|  | MXL -- Mexican | 3.78 | 6,026 | |
|  | PEL -- Peruvian | 3.75 | 5,623 | |
|  | PUR -- Puerto Rican | 3.86 | 7,244 | |
| East Asian | CDX -- Chinese Dai | 3.37 | 2,344 | |
|  | CHB -- Han Chinese in Bejing | 3.34 | 2,188 | |
|  | CHS -- Southern Han Chinese | 3.32 | 2,089 | |
|  | JPT -- Japanese | 3.31 | 2,042 | |
|  | KHV -- Kinh | 3.4 | 2,512 | |
| European | CEU -- Utah residents | 3.42 | 2,630 | |
|  | FIN -- Finnish | 3.47 | 2,951 | |
|  | GBR -- British | 3.41 | 2,570 | |
|  | IBS -- Iberian | 3.59 | 3,890 | |
|  | TSI -- Toscani | 3.47 | 2,951 | |
| South Asian | BEB -- Bengali | 3.42 | 2,630 | |
|  | GIH -- Gujarati Indian | 3.44 | 2,754 | |
|  | ITU -- Indian Telugu | 3.43 | 2,692 | |
|  | PJL -- Punjabi | 3.43 | 2,692 | |
|  | STU -- Sri Lankan Tamil | 3.44 | 2,754 | |
