# Supplementary material for: An estimator of first coalescent time reveals selection on young variants and large heterogeneity in rare allele ages among human populations
Source: PLoS Genet. 2019 Aug 19;15(8):e1008340. doi: 10.1371/journal.pgen.1008340 (PMC6715256; doi:10.1371/journal.pgen.1008340)
Supplement: S4 Table — (DOCX) [file pgen.1008340.s004.docx]

| Number of chromosomes | Number of SNPS | $msh (s$econds) | $\hat{t}_{c}$ (seconds) |
| --- | --- | --- | --- |
| 7242 | 568,132 | 1499 | 163 |
| 7242 | 56,800 | 137 | 18 |
| 724 | 568,132 | 142 | 46 |
| 724 | 56,800 | 15 | 4 |
