# Supplementary material for: An estimator of first coalescent time reveals selection on young variants and large heterogeneity in rare allele ages among human populations
Source: PLoS Genet. 2019 Aug 19;15(8):e1008340. doi: 10.1371/journal.pgen.1008340 (PMC6715256; doi:10.1371/journal.pgen.1008340)
Supplement: S5 Table — Data were simulated to generate sample of 100 and 1000 chromosomes under each of two recombination rates (1 × 10^−9^ and 1 × 10^−8^), and under each of two demographic models: one with a constant size of N = 1 × 10^4^; and one with an historical size of N = 1 × 10^4^ followed by exponential growth over the last 200 generations to a final size of N = 5 × 10^5^. Two versions of the tc estimator were applied to all alleles that occurred in each of the 8 simulations, one that assumed a constant N = 1 × 10^4^ and a second that assumed a constant N = 5 × 10^5^. Results are the average difference in log10(t^c) values and Pearson’s r statistic between the log10(t^c) values. (DOCX) [file pgen.1008340.s005.docx]

| sample size | demography | recombination rate | average difference | $r$ |
| --- | --- | --- | --- | --- |
| 100 | constant | $1\times{10}^{-9}$ | 0.07 | 1 |
|  |  | $1\times{10}^{-8}$ | 0.08 | 1 |
|  | recent growth | $1\times{10}^{-9}$ | 0.07 | 1 |
|  |  | $1\times{10}^{-8}$ | 0.08 | 1 |
| 1000 | constant | $1\times{10}^{-9}$ | 0.05 | 1 |
|  |  | $1\times{10}^{-8}$ | 0.06 | 1 |
|  | recent growth | $1\times{10}^{-9}$ | 0.07 | 1 |
|  |  | $1\times{10}^{-8}$ | 0.08 | 1 |
