# Supplementary material for: An estimator of first coalescent time reveals selection on young variants and large heterogeneity in rare allele ages among human populations
Source: PLoS Genet. 2019 Aug 19;15(8):e1008340. doi: 10.1371/journal.pgen.1008340 (PMC6715256; doi:10.1371/journal.pgen.1008340)
Supplement: S6 Table — (DOCX) [file pgen.1008340.s006.docx]

|  | coefficient |
| --- | --- |
| Intercept | 3.9055 |
| Population [ASW] | -0.0235 |
| Population [BEB] | -0.3798 |
| Population [CDX] | -0.3649 |
| Population[CEU] | -0.3493 |
| Population[CHB] | -0.4069 |
| Population[CHS] | -0.4365 |
| Population[CLM] | 0.0705 |
| Population[ESN] | 0.0403 |
| Population[FIN] | -0.2901 |
| Population[GBR] | -0.37 |
| Population[GIH] | -0.3552 |
| Population[GWD] | 0.0572 |
| Population[IBS] | -0.1719 |
| Population[ITU] | -0.3559 |
| Population[JPT] | -0.3997 |
| Population[KHV] | -0.334 |
| Population[LWK] | 0.0573 |
| Population[MSL] | 0.0169 |
| Population[MXL] | 0.054 |
| Population[PEL] | 0.1356 |
| Population[PJL] | -0.3624 |
| Population[PUR] | 0.0536 |
| Population[STU] | -0.3476 |
| Population[TSI] | -0.284 |
| Population[YRI] | 0.0222 |
| PC[True] | -0.0158 |
| Private[True] | -0.5196 |
| PC[True]xPopulation[ASW] | 0.0002 |
| PC[True]xPopulation[BEB] | 0.0008 |
| PC[True]xPopulation[CDX] | -0.0214 |
| PC[True]xPopulation[CEU] | 0.0191 |
| PC[True]xPopulation[CHB] | 0.0208 |
| PC[True]xPopulation[CHS] | 0.0029 |
| PC[True]xPopulation[CLM] | 0.0119 |
| PC[True]xPopulation[ESN] | 0.0041 |
| PC[True]xPopulation[FIN] | 8.58E-05 |
| PC[True]xPopulation[GBR] | 0.0122 |
| PC[True]xPopulation[GIH] | -0.0114 |
| PC[True]xPopulation[GWD] | 0.0078 |
| PC[True]xPopulation[IBS] | 0.0127 |
| PC[True]xPopulation[ITU] | 0.0044 |
| PC[True]xPopulation[JPT] | -0.002 |
| PC[True]xPopulation[KHV] | 0.0043 |
| PC[True]xPopulation[LWK] | 0.0098 |
| PC[True]xPopulation[MSL] | 0.0107 |
| PC[True]xPopulation[MXL] | 0.0053 |
| PC[True]xPopulation[PEL] | 0.0114 |
| PC[True]xPopulation[PJL] | 0.0025 |
| PC[True]xPopulation[PUR] | 0.0071 |
| PC[True]xPopulation[STU] | -0.0063 |
| PC[True]xPopulation[TSI] | -0.0035 |
| PC[True]xPopulation[YRI] | -0.0032 |
| PC[True]xPrivate[True] | 0.0081 |
| Population[ASW]xPrivate[True] | 0.0389 |
| Population[BEB]xPrivate[True] | 0.1399 |
| Population[CDX]xPrivate[True] | 0.0144 |
| Population[CEU]xPrivate[True] | -0.0177 |
| Population[CHB]xPrivate[True] | 0.0654 |
| Population[CHS]xPrivate[True] | 0.0849 |
| Population[CLM]xPrivate[True] | -0.3428 |
| Population[ESN]xPrivate[True] | -0.1254 |
| Population[FIN]xPrivate[True] | -0.1713 |
| Population[GBR]xPrivate[True] | -0.0236 |
| Population[GIH]xPrivate[True] | 0.0389 |
| Population[GWD]xPrivate[True] | -0.1391 |
| Population[IBS]xPrivate[True] | -0.1553 |
| Population[ITU]xPrivate[True] | 0.0918 |
| Population[JPT]xPrivate[True] | 0.0372 |
| Population[KHV]xPrivate[True] | 0.0348 |
| Population[LWK]xPrivate[True] | 0.1349 |
| Population[MSL]xPrivate[True] | -0.022 |
| Population[MXL]xPrivate[True] | -0.3948 |
| Population[PEL]xPrivate[True] | -0.5628 |
| Population[PJL]xPrivate[True] | 0.1071 |
| Population[PUR]xPrivate[True] | -0.2845 |
| Population[STU]xPrivate[True] | 0.0976 |
| Population[TSI]xPrivate[True] | -0.0282 |
| Population[YRI]xPrivate[True] | -0.1316 |
