# Supplementary figures and images for: An estimator of first coalescent time reveals selection on young variants and large heterogeneity in rare allele ages among human populations
Source: PLoS Genet. 2019 Aug 19;15(8):e1008340. doi: 10.1371/journal.pgen.1008340 (PMC6715256; doi:10.1371/journal.pgen.1008340)

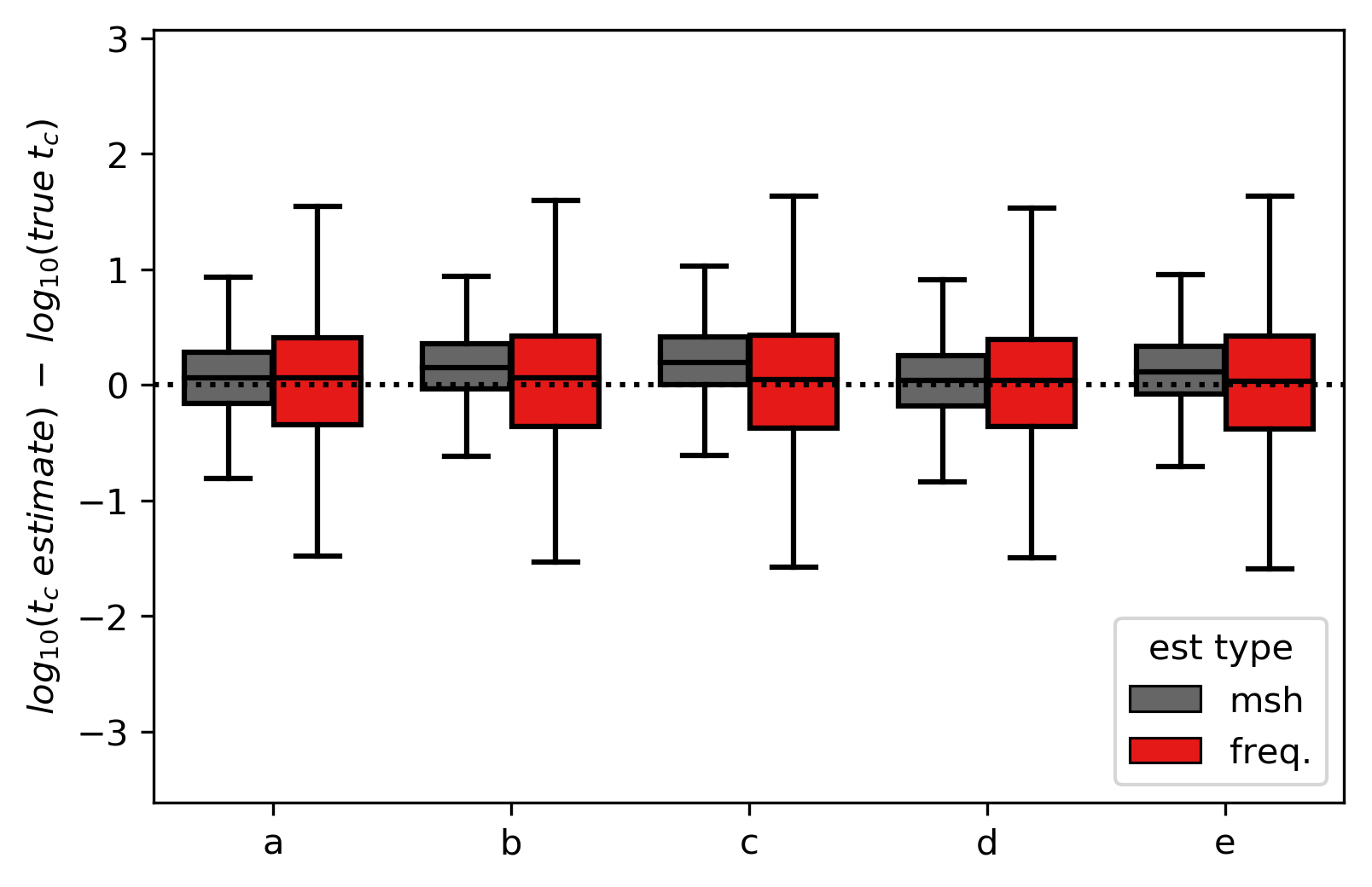

Supplement: S1 Fig — Using the data from the simulations shown in Fig 2, these boxplots show the increase in precision in estimating tc from msh compared to the best possible estimator operating on variant frequency. Cells a-c represent samples of n = 1000. Cells d-e represent samples of n = 100. a and d include all variants of frequency ≤ 10%, b and e represent variants of frequency ≤ 1%, and c is only variants found at 0.1% frequency. (TIF) [file pgen.1008340.s007.tif]

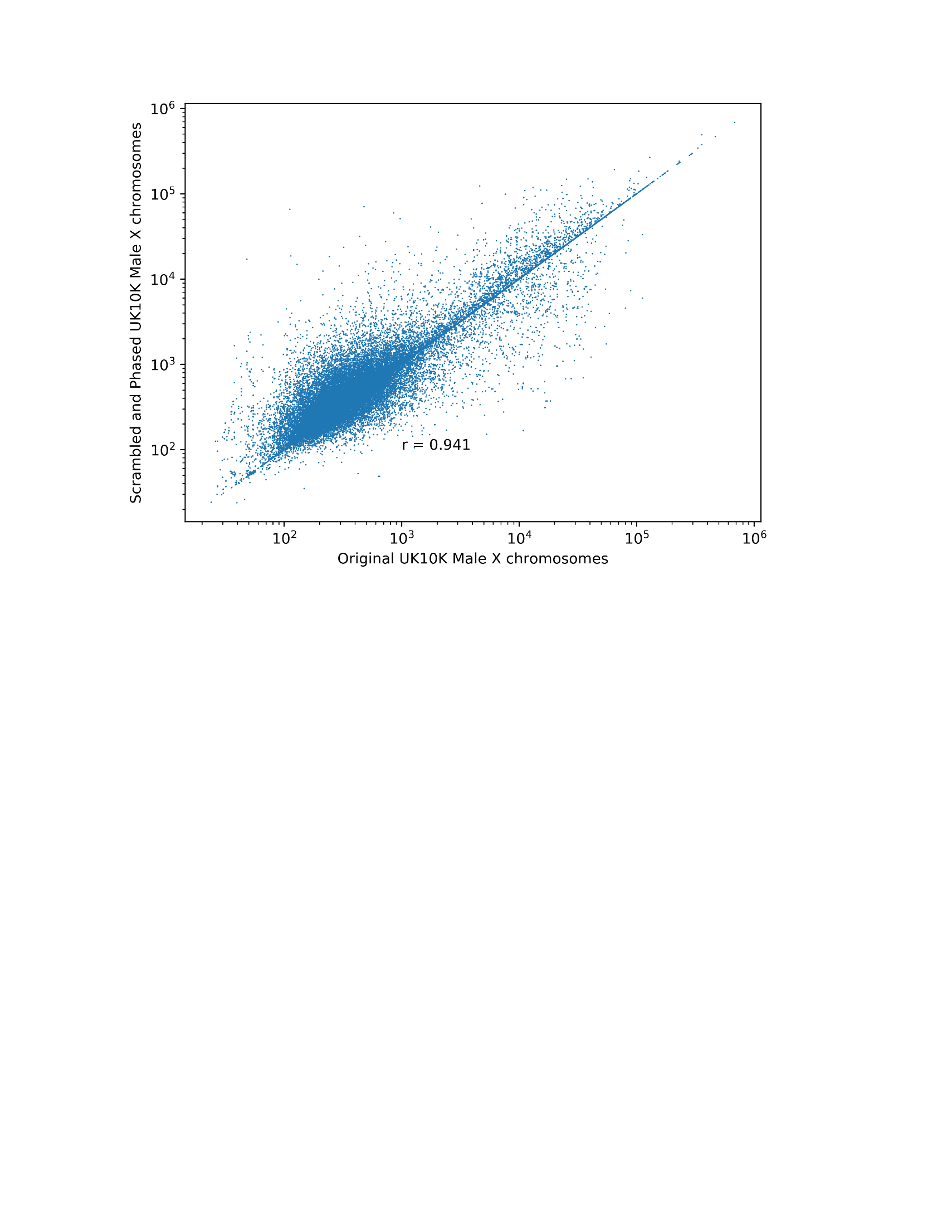

Supplement: S2 Fig — The X axis shows values for a set of original X chromosomes, and the Y chromosome shows values for the same data after randomization to simulate 50 (diploid) pairs of unphased chromosomes, followed by application of phasing software and singleton phasing as described in the manuscript. To generate rephrased data, 100 chromosomes were randomly paired, and heterozygous positions were randomized to simulate a diploid, short-read, assembly, and then phased using SHAPEIT [37]. We used a mutation rate of 10−8 per base pair and the deCODE genetic map [31, 38]. (TIF) [file pgen.1008340.s008.tif]

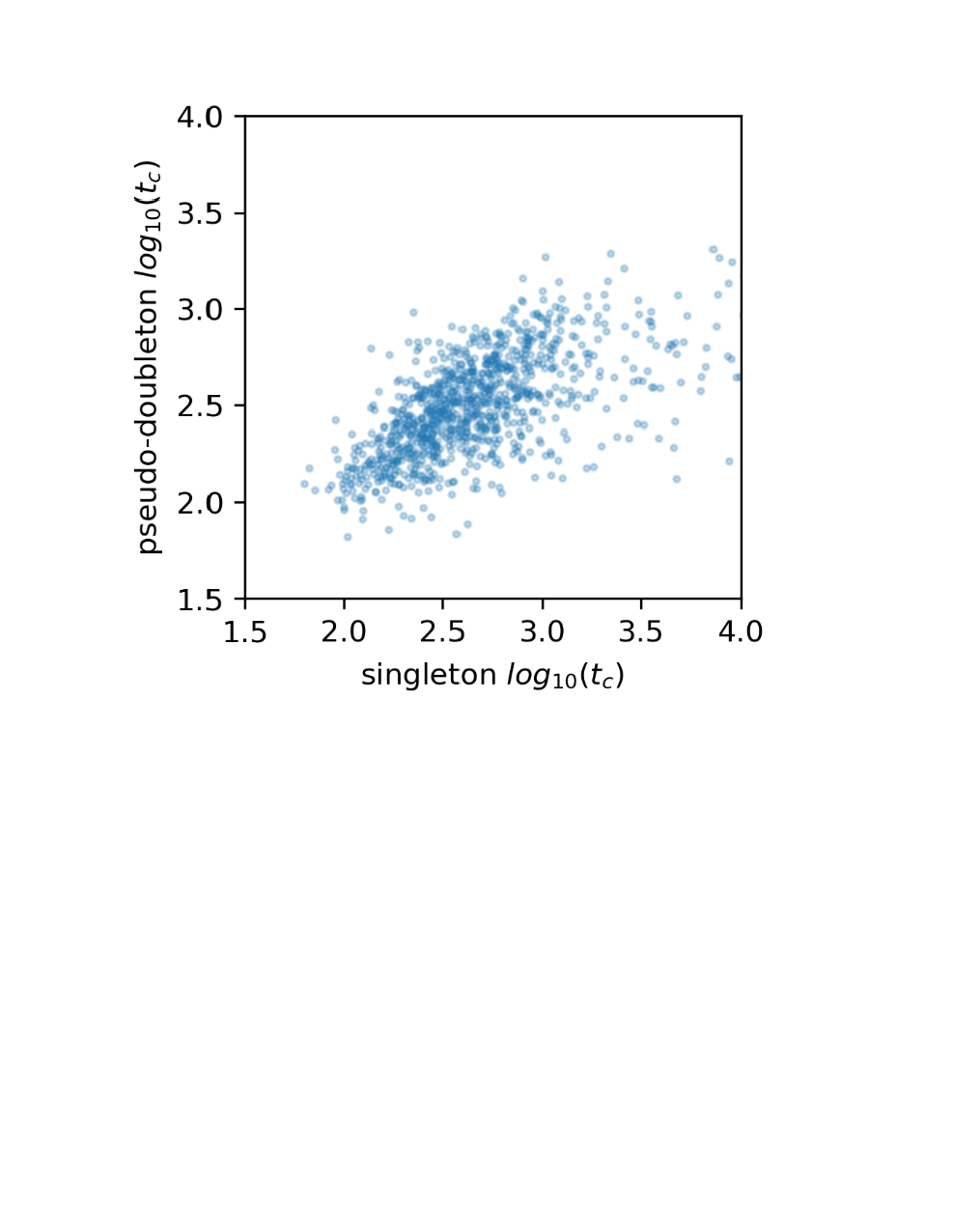

Supplement: S3 Fig — Shown is the case in which a locus harbors both a singleton variant and a false-positive SNP call. 1000 doubleton variants are generated by randomly selecting a singleton SNP in the UK10K sample, and then selecting an additional chromosome at random and assigning to it the derived allele. We compare the original singleton tc estimate with the tc estimate of the artificially created doubleton variant. The addition of a derived allele that is identical by state but not identical by descent does not produce radically deviant estimates. True double mutations are expected to behave in a similar manner, though are more likely to be found on longer branches than randomly chosen branches used for this figure. (TIF) [file pgen.1008340.s009.tif]

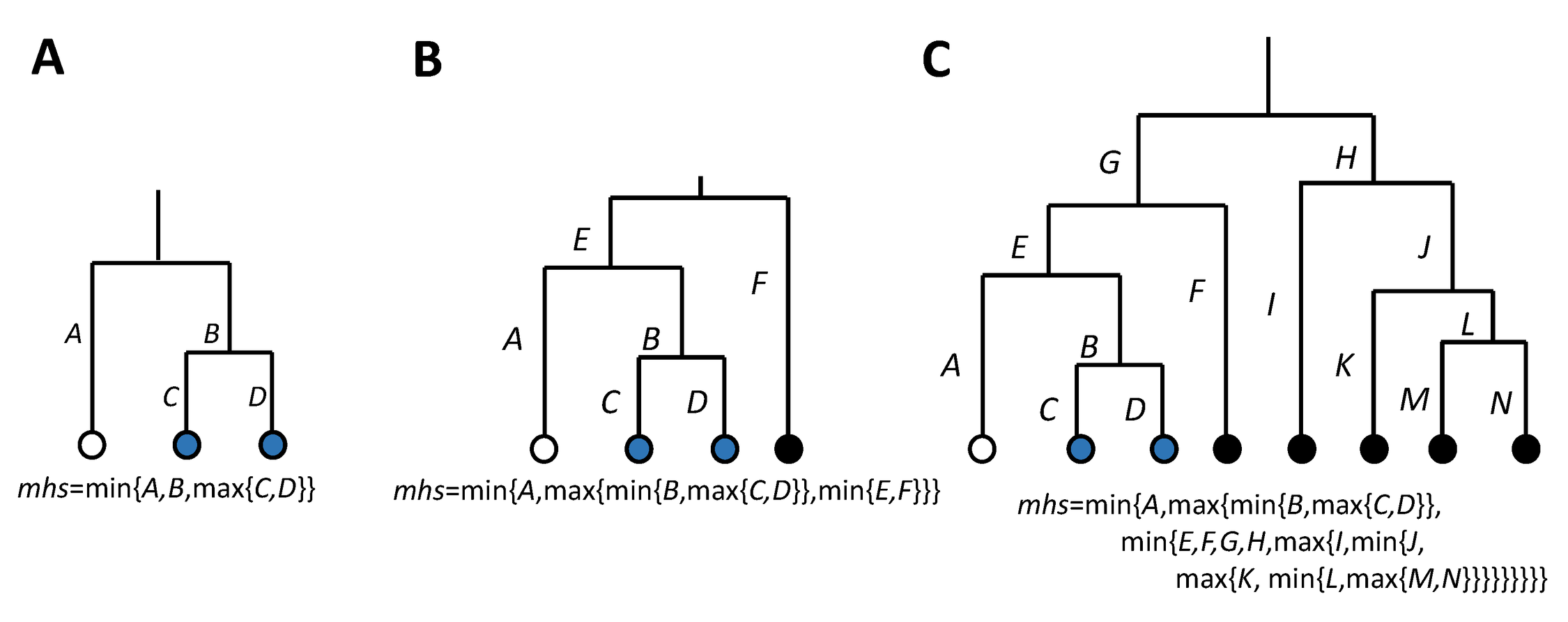

Supplement: S4 Fig — Panels A, B and C show three different sample sizes. Within each panel, every edge has a value in italics (A, B, C, D etc) that is the distance from the focal base to the closest mutation to that base that occurred on that edge. Below each figure is given the maximum shared haplotype (msh) value as a function of the distances to the mutations on each edge. (TIF) [file pgen.1008340.s010.tif]

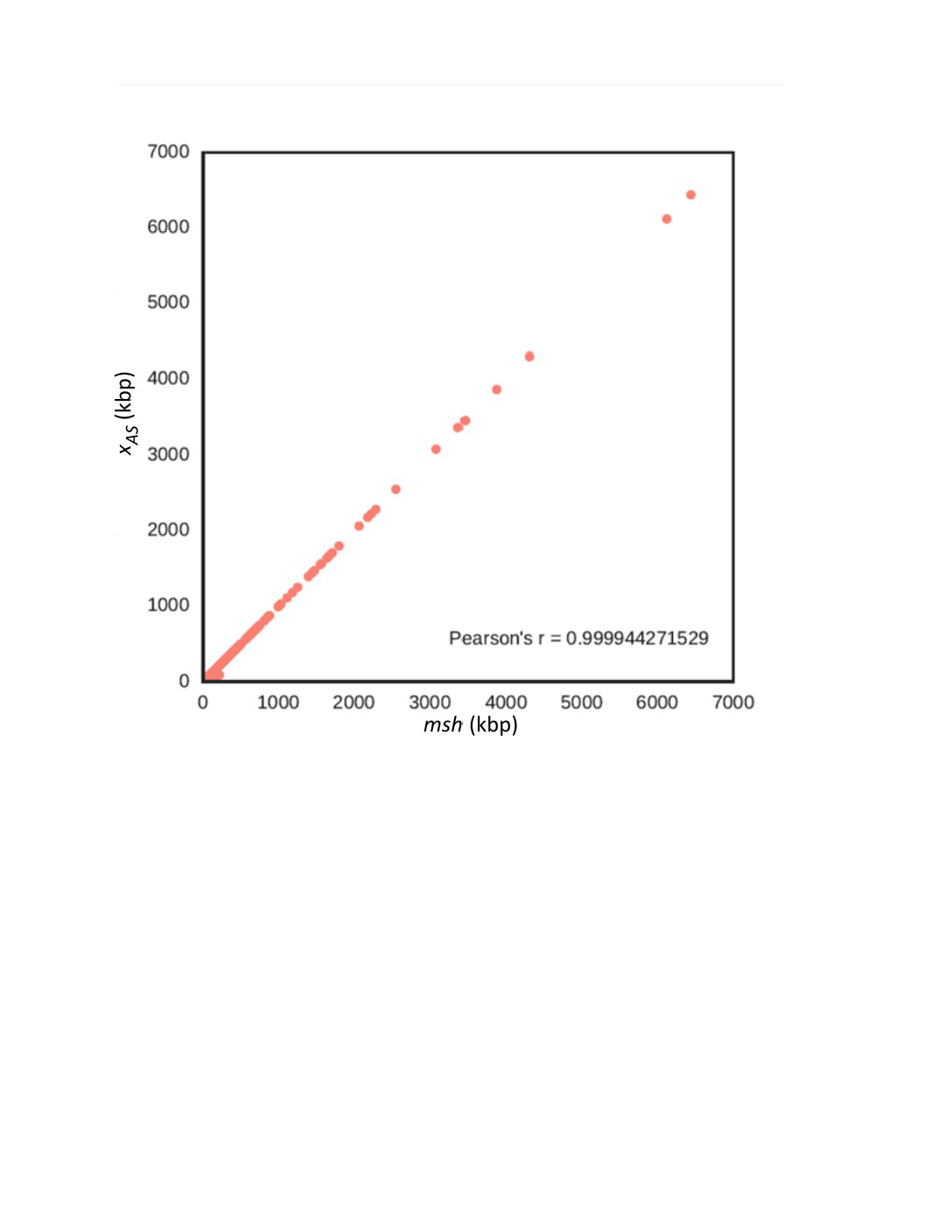

Supplement: S5 Fig — xAS are plotted against the corresponding msh values as determined in a set of simulated data. Coalescent simulations (104 independent simulations) were conducted for a sample of 100 chromosomes drawn from a constant-sized diploid population of N = 106, with a per base mutation rate of 2 × 10^−8^, and no recombination using msprime [35]. For each simulated data set we measured msh of a singleton variant and the longest maximum shared haplotype determined by considering only events on the external branch immediately ancestral to the singleton variant and its first sister branch (approximate msh). (TIF) [file pgen.1008340.s011.tif]

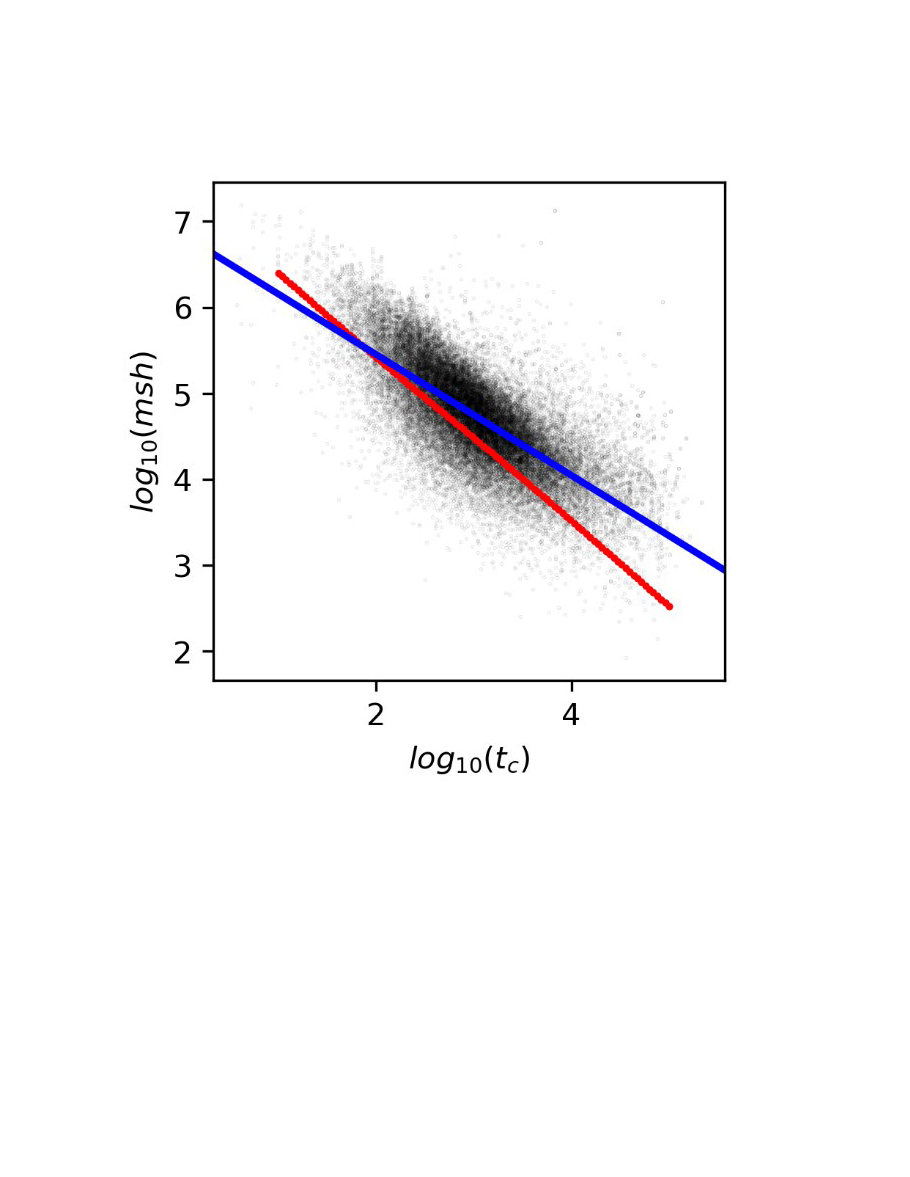

Supplement: S6 Fig — Black dots represent observed msh, values from singleton variants from n = 100 chromosomes sampled from a simulated constant-sized population of N = 1 × 10^4^ individuals with per-base mutation and recombination rates of μ = ρ = 1 × 10^−8^ as a function of their true tc values. The red line is the expected value of a one-direction msh tract as a function of tc, given the same parameter values, obtained by integration of Eq 4 (slope: -0.961, intercept: 7.353). The blue line is the linear regression of log10(msh) on log10(tc) (slope: -0.703, intercept: 6.858). (TIF) [file pgen.1008340.s012.tif]

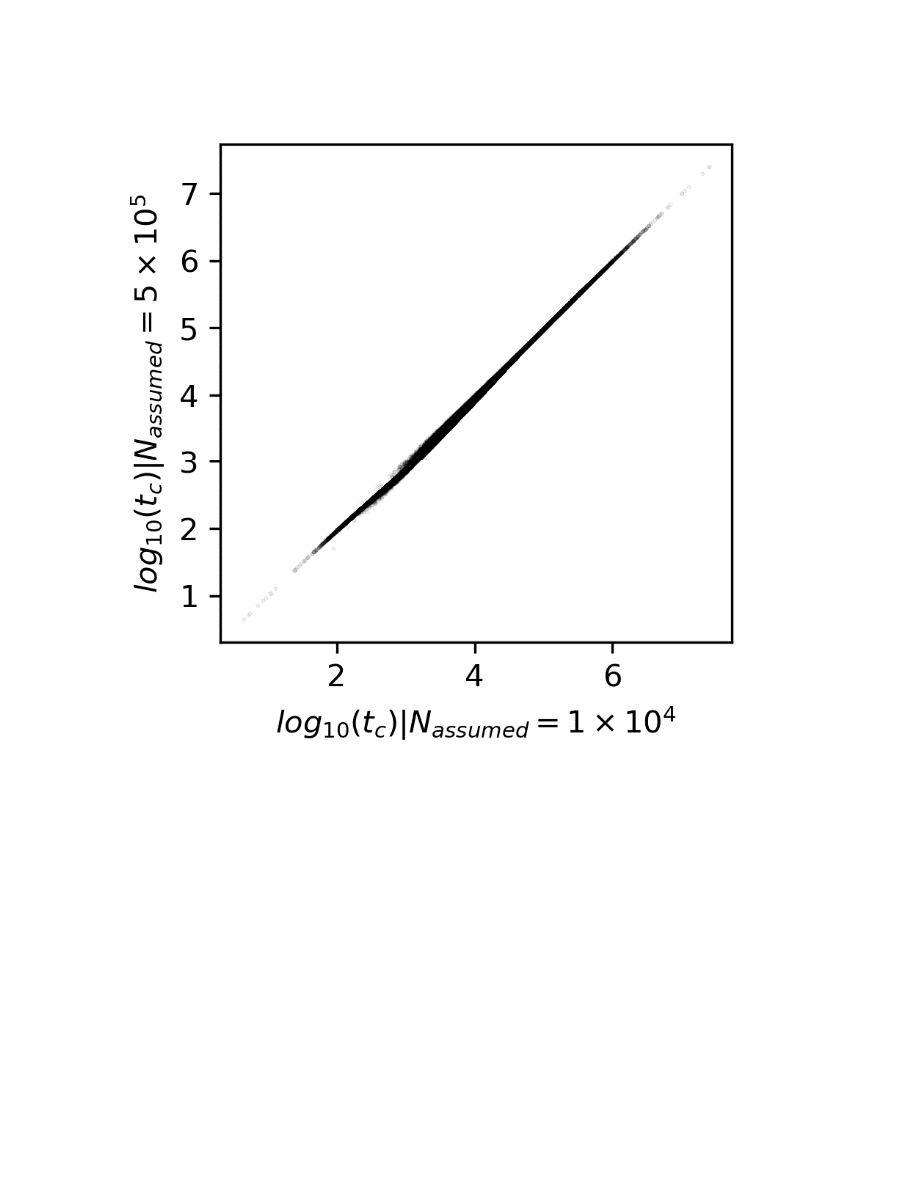

Supplement: S7 Fig — Variants from 100 chromosomes simulated from a population with an historic size of N = 1 × 10^4^ and exponential growth over the last 200 generations to a size of N = 5 × 10^5^ at the time of sampling (recent growth) have tc estimated under extreme assumptions of a constant demography of N = 1 × 10^4^ and a constant demography of N = 5 × 10^5^. (TIF) [file pgen.1008340.s013.tif]

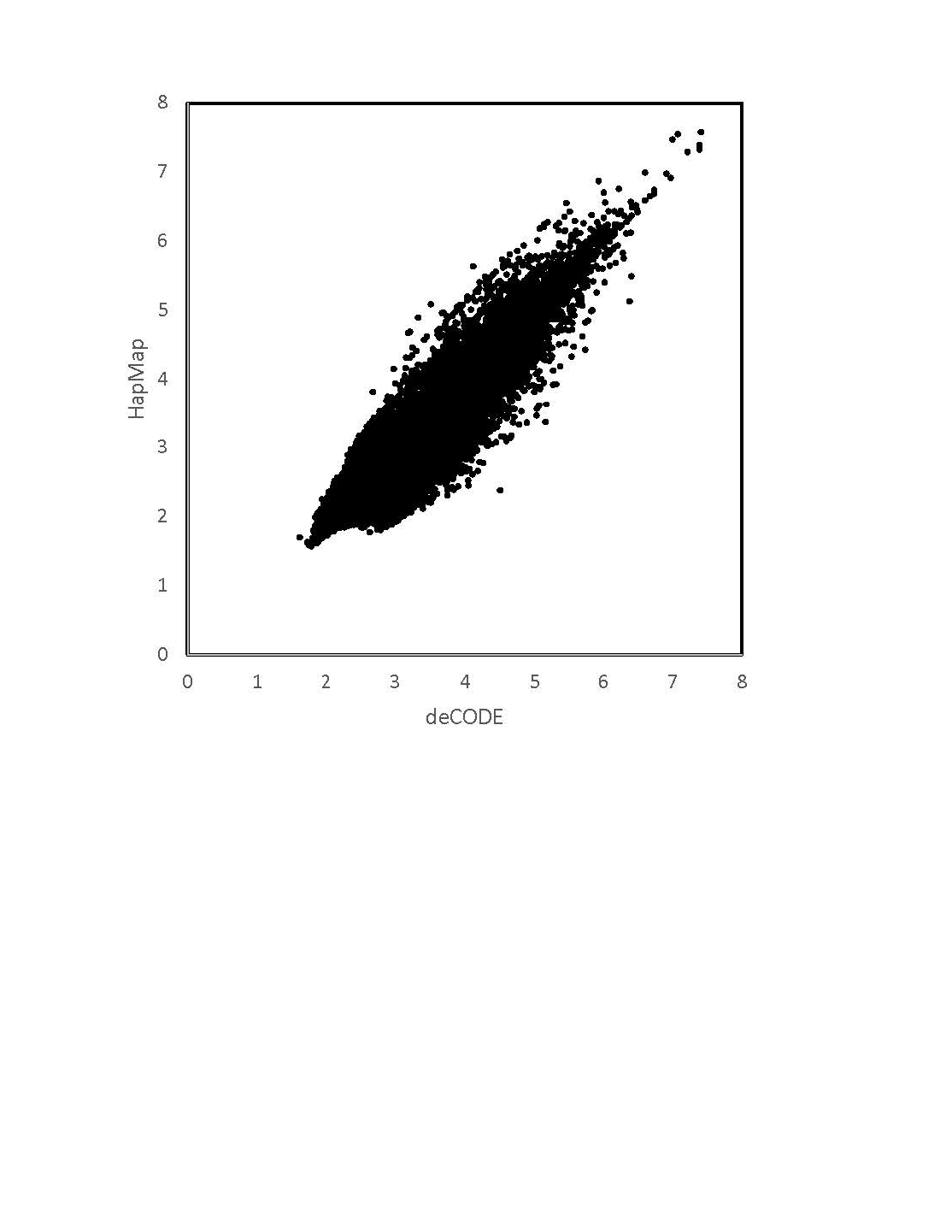

Supplement: S8 Fig — Log10(t^c) values for 88,651 low frequency UK10K alleles from chromosome 22 with counts between 2 and 10, inclusive. Estimates were generated using the HapMap [26] genetic map, which has a total length of 80 centimorgans, and the deCODE map [31], which has a total length of 55 centimorgans. Pearson’s correlation coefficient is 0.929. (TIF) [file pgen.1008340.s014.tif]

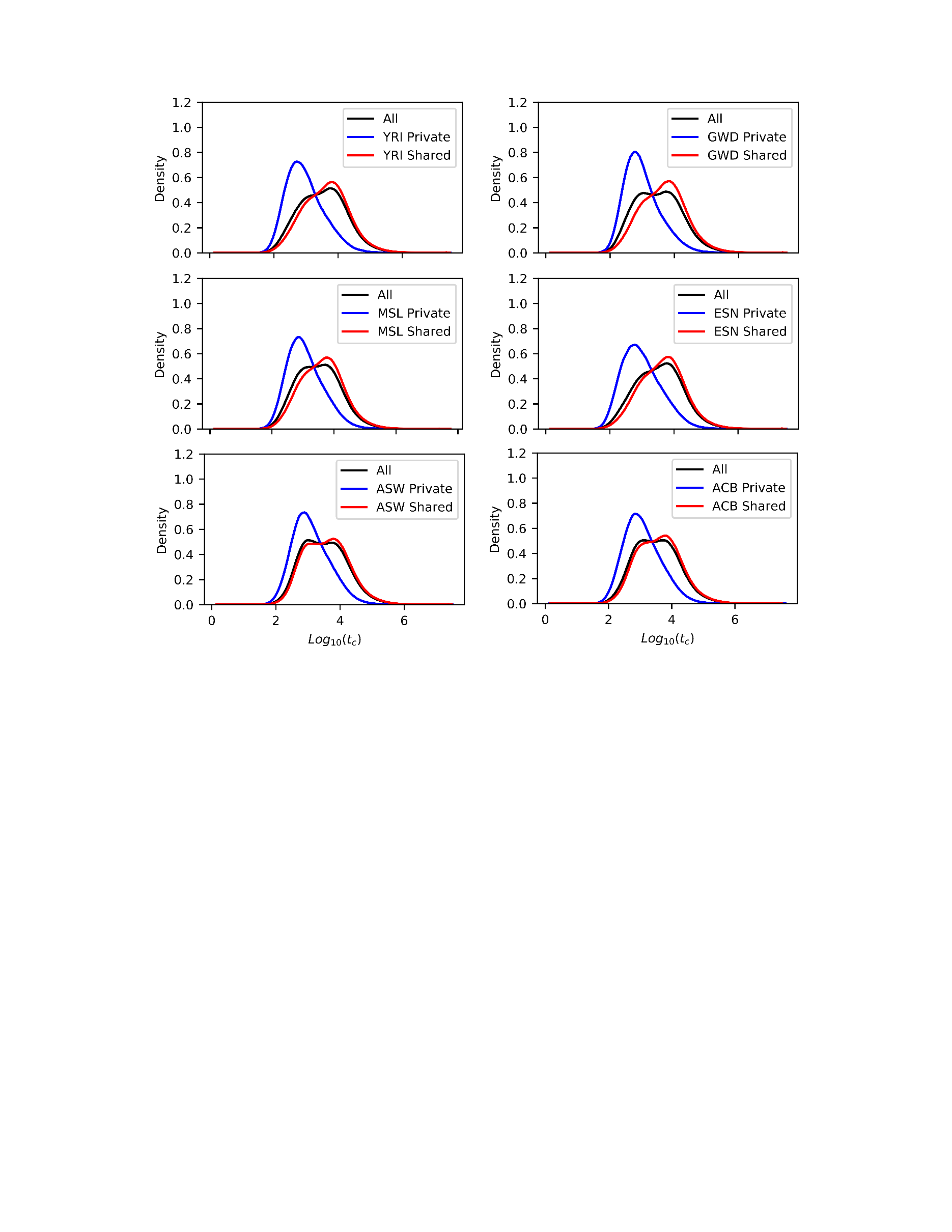

Supplement: S9 Fig — Distributions are shown for private alleles and alleles that are also found in other populations, and for both. (TIF) [file pgen.1008340.s015.tif]

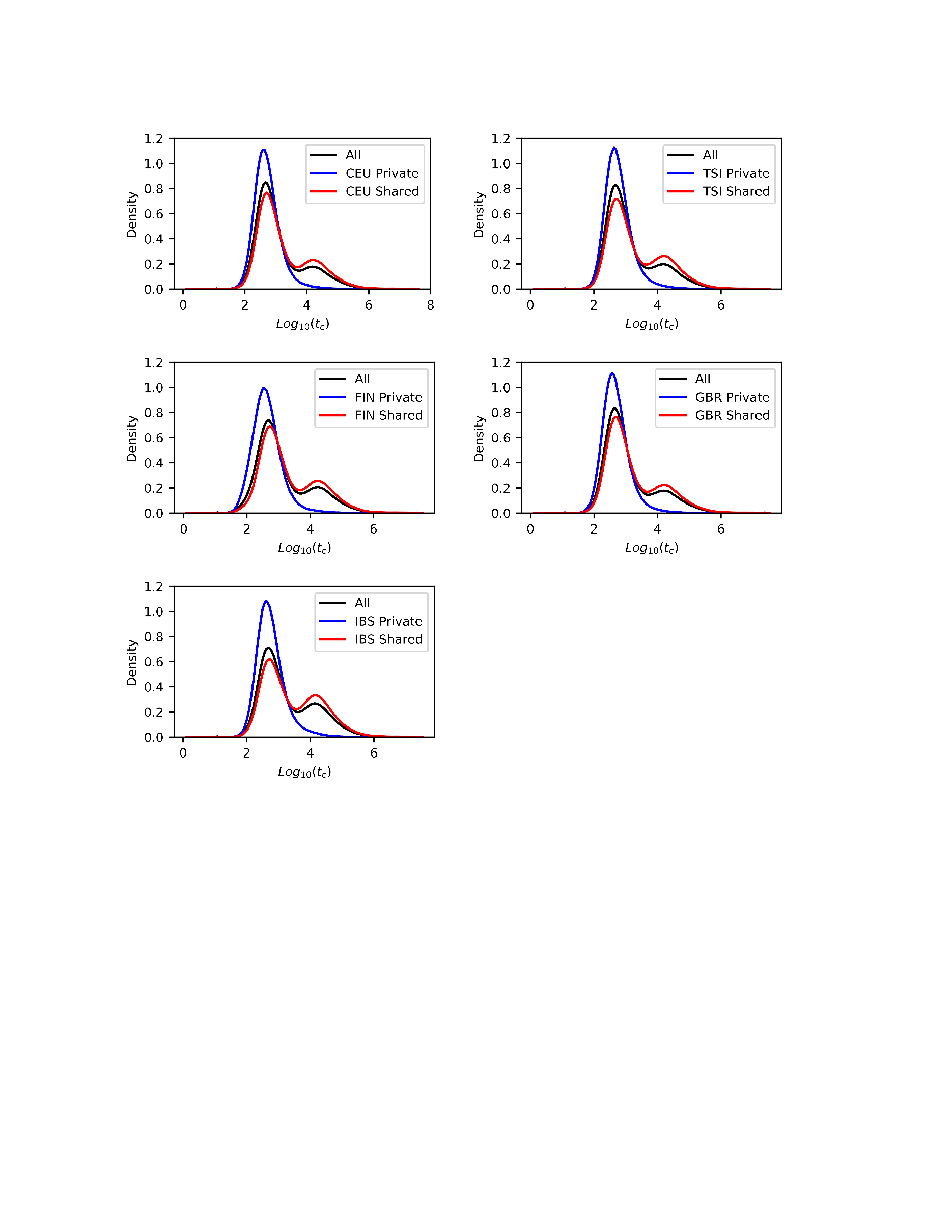

Supplement: S10 Fig — Distributions are shown for private alleles and alleles that are also found in other populations, and for both. (TIF) [file pgen.1008340.s016.tif]

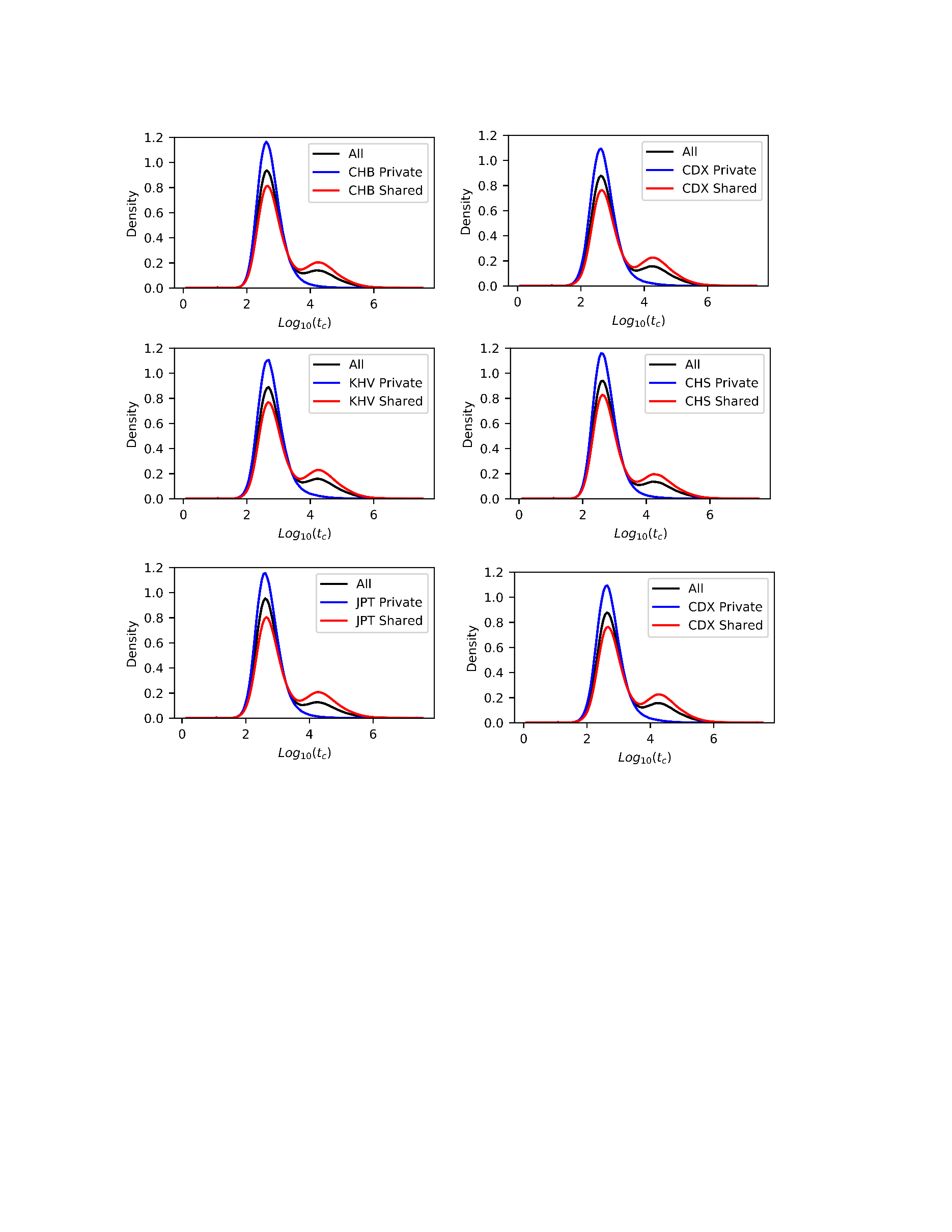

Supplement: S11 Fig — Distributions are shown for private alleles and alleles that are also found in other populations, and for both. (TIF) [file pgen.1008340.s017.tif]

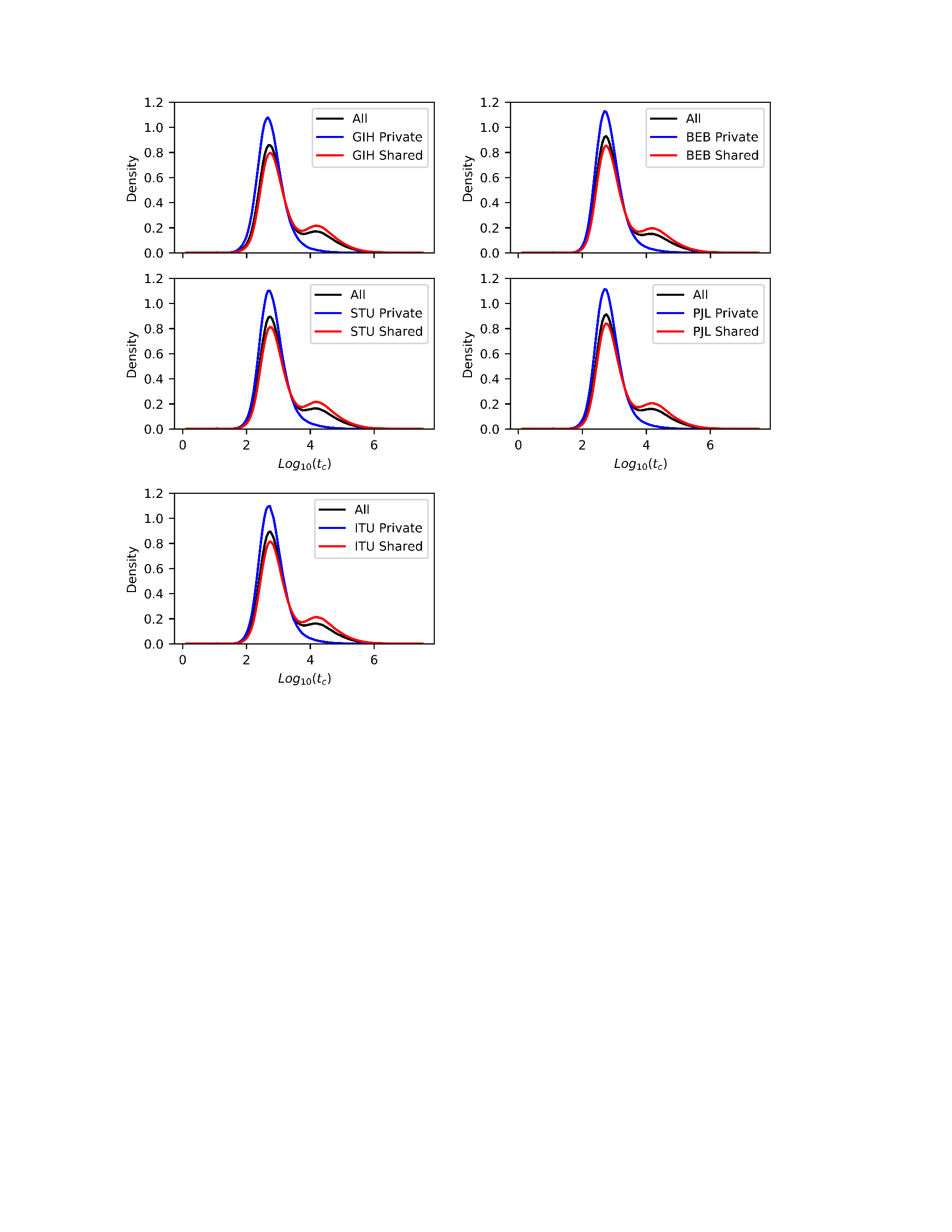

Supplement: S12 Fig — Distributions are shown for private alleles and alleles that are also found in other populations, and for both. (TIF) [file pgen.1008340.s018.tif]

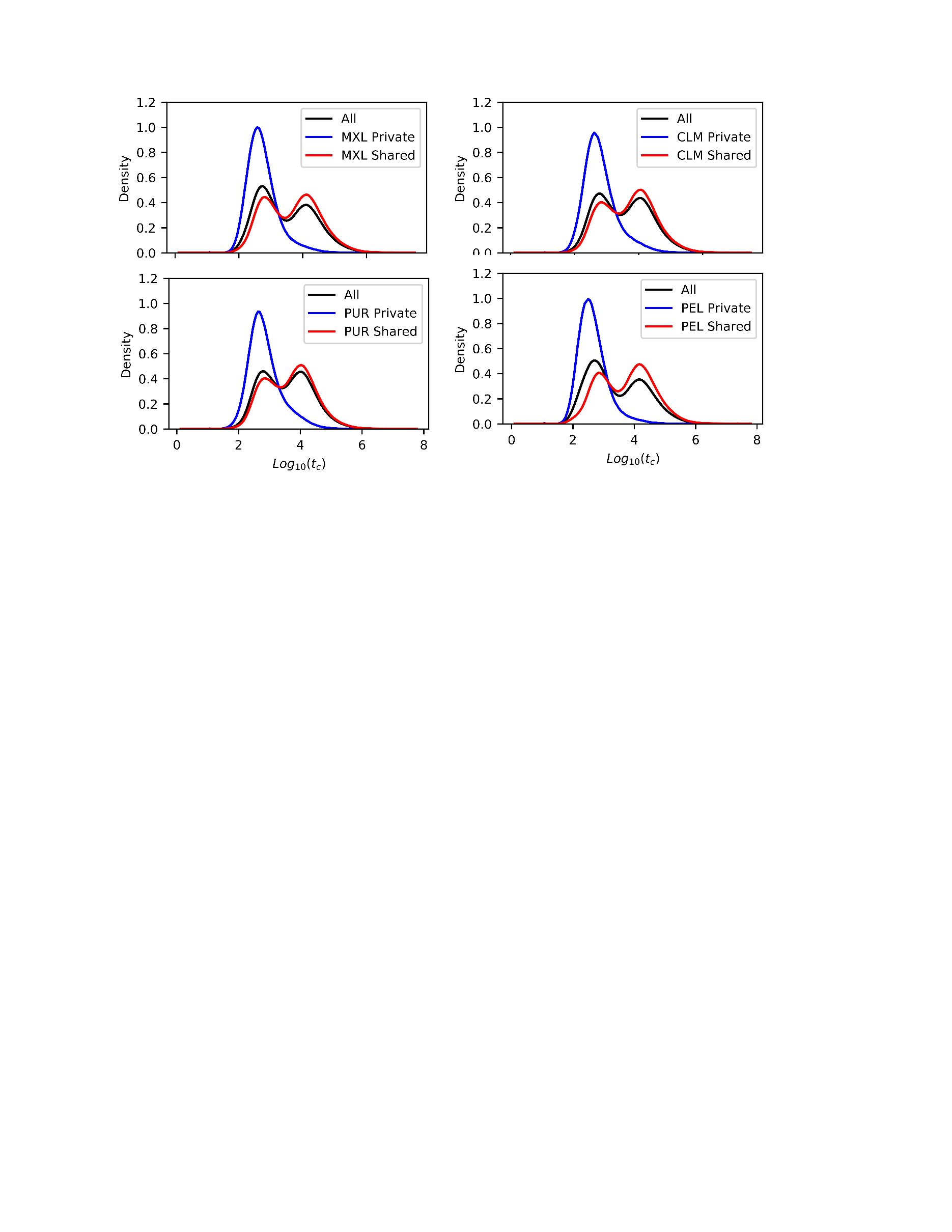

Supplement: S13 Fig — Distributions are shown for private alleles and alleles that are also found in other populations, and for both. (TIF) [file pgen.1008340.s019.tif]

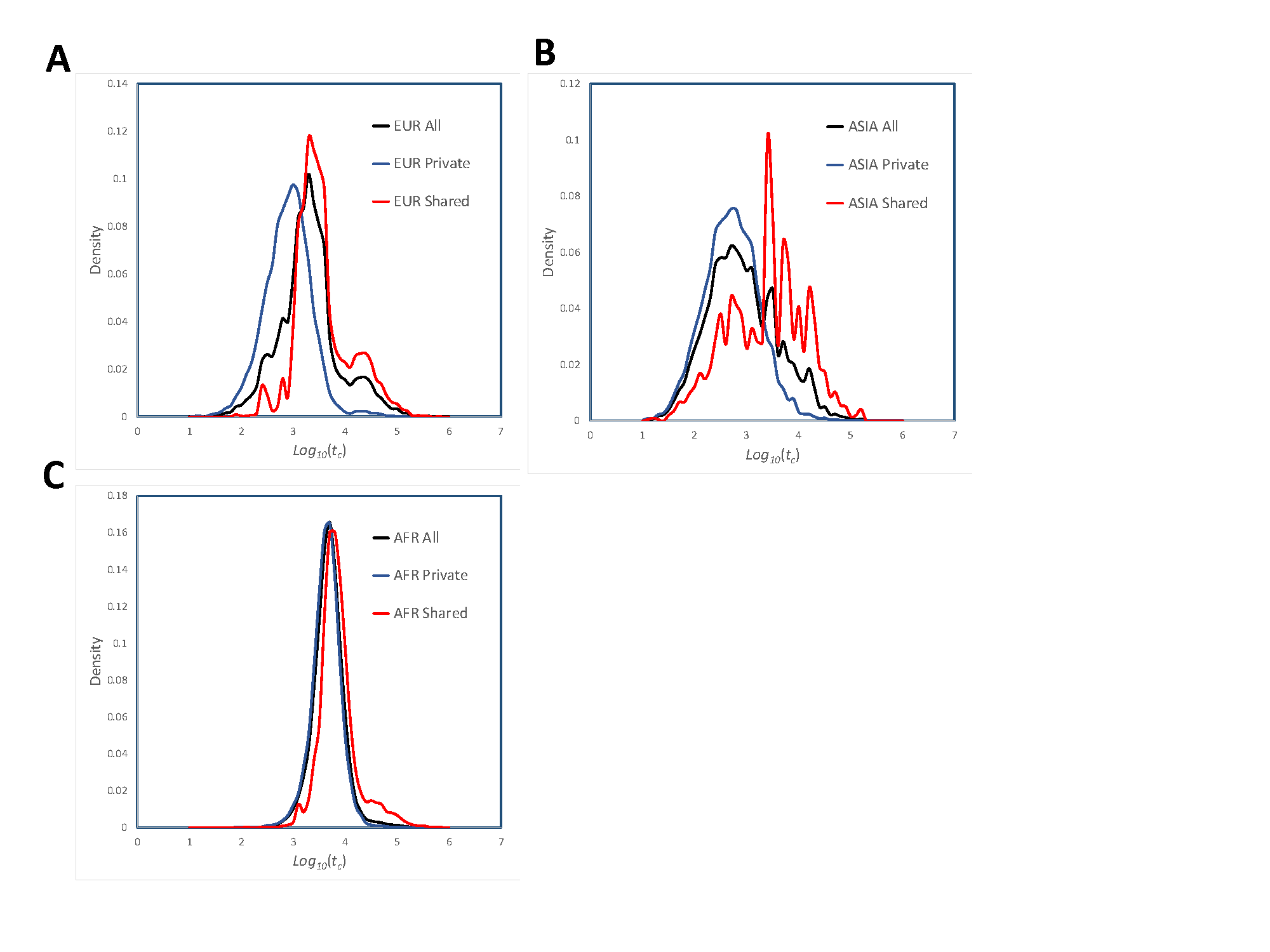

Supplement: S14 Fig — Following the parameter estimates of Gutenkunst et al., [24], 100 chromosomes of length 109 bases for each population were simulated using SCRM [39], with per base mutation rates of 1e-8 for both mutation and recombination. Distributions are shown for Europe (A), Asia (B) and Africa (C) for private alleles and alleles that are also found in other populations, and for both. (TIF) [file pgen.1008340.s020.tif]

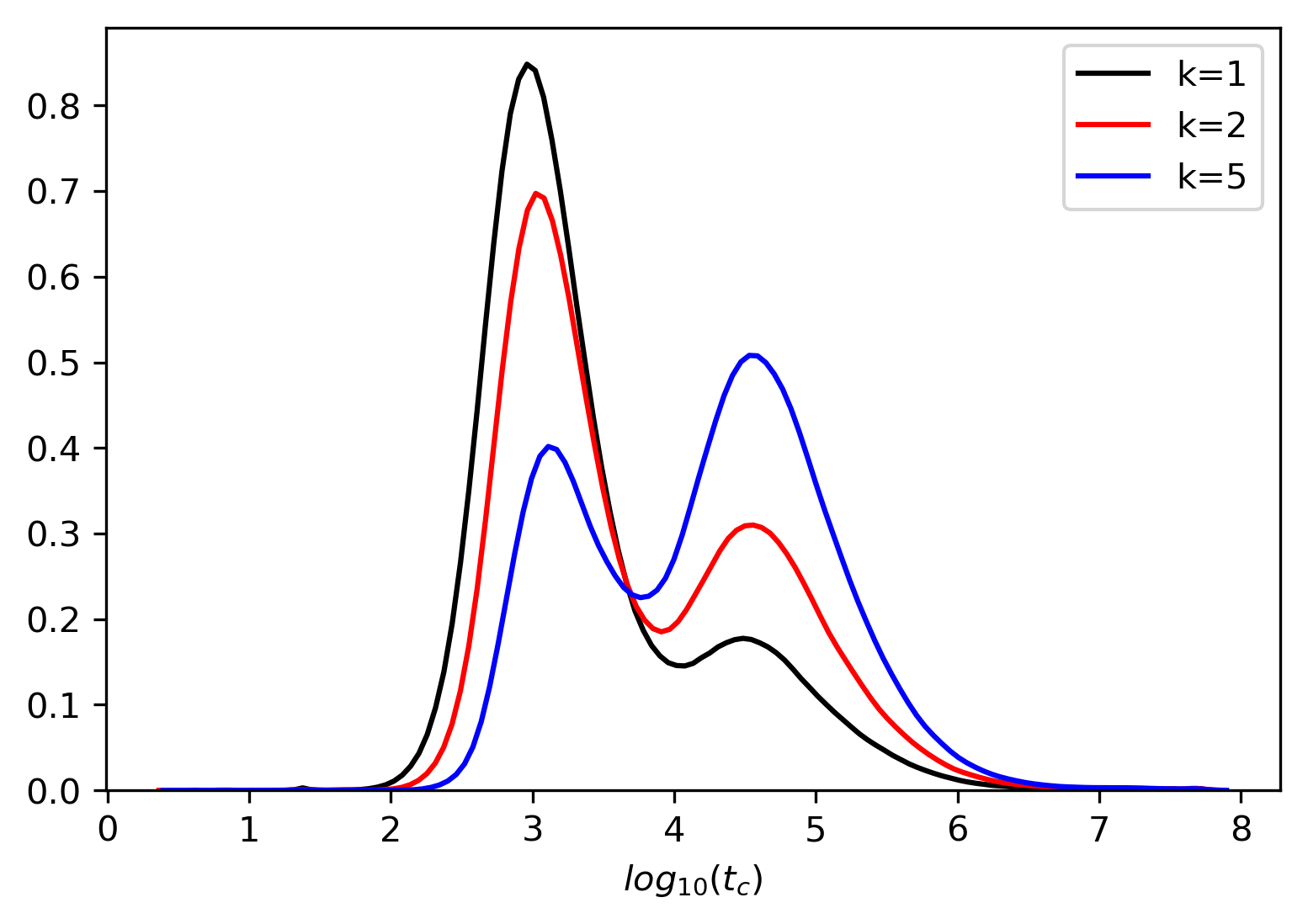

Supplement: S15 Fig — (TIF) [file pgen.1008340.s021.tif]
